# Assessment of Oxygen Supply-Demand Imbalance and Outcomes Among Patients With Type 2 Myocardial Infarction: A Secondary Analysis of the High-STEACS Cluster Randomized Clinical Trial

**DOI:** 10.1001/jamanetworkopen.2022.20162

**Published:** 2022-07-11

**Authors:** Anda Bularga, Caelan Taggart, Filip Mendusic, Dorien M. Kimenai, Ryan Wereski, Matthew T. H. Lowry, Kuan K. Lee, Amy V. Ferry, Stacey S. Stewart, David A. McAllister, Anoop S. V. Shah, Atul Anand, David E. Newby, Nicholas L. Mills, Andrew R. Chapman

**Affiliations:** 1 British Heart Foundation Centre for Cardiovascular Science, University of Edinburgh, Edinburgh, United Kingdom; 2 Usher Institute, University of Edinburgh, Edinburgh, United Kingdom; 3 Institute of Health and Wellbeing, University of Glasgow, Glasgow, United Kingdom; 4 Department of Non-communicable Disease, London School of Hygiene and Tropical Medicine, London, United Kingdom; 5 Department of Cardiology, Imperial College Healthcare NHS Trust, London, United Kingdom

## Abstract

**Importance:**

Type 2 myocardial infarction occurs owing to multiple factors associated with myocardial oxygen supply-demand imbalance, which may confer different risks of adverse outcomes.

**Objective:**

To evaluate the prevalence and outcomes of different factors associated with oxygen supply-demand imbalance among patients with type 2 myocardial infarction.

**Design, Setting, and Participants:**

In this secondary analysis of a stepped-wedge, cluster randomized clinical trial conducted at 10 secondary and tertiary care hospitals in Scotland, 6096 patients with an adjudicated diagnosis of type 1 or type 2 myocardial infarction from June 10, 2013, to March 3, 2016, were identified, and the findings were reported on August 28, 2018. The trial enrolled consecutive patients with suspected acute coronary syndrome. The diagnosis of myocardial infarction was adjudicated according to the Fourth Universal Definition of Myocardial Infarction and the primary factor associated with oxygen supply-demand imbalance in type 2 myocardial infarction was defined. This secondary analysis was not prespecified. Statistical analysis was performed from July 7 to 30, 2020.

**Intervention:**

Implementation of a high-sensitivity cardiac troponin I assay.

**Main Outcomes and Measures:**

All-cause death at 1 year according to the factors associated with oxygen supply-demand imbalance among patients with type 2 myocardial infarction.

**Results:**

Of 6096 patients (2602 women [43%]; median age, 70 years [IQR, 58-80 years]), 4981 patients had type 1 myocardial infarction, and 1115 patients had type 2 myocardial infarction. The most common factor associated with oxygen supply-demand imbalance was tachyarrhythmia (616 of 1115 [55%]), followed by hypoxemia (219 of 1115 [20%]), anemia (95 of 1115 [9%]), hypotension (89 of 1115 [8%]), severe hypertension (61 of 1115 [5%]), and coronary mechanisms (35 of 1115 [3%]). At 1 year, all-cause mortality occurred for 15% of patients (720 of 4981) with type 1 myocardial infarction and 23% of patients (285 of 1115) with type 2 myocardial infarction. Compared with patients with type 1 myocardial infarction, those with type 2 myocardial infarction owing to hypoxemia (adjusted odds ratio [aOR], 2.35; 95% CI, 1.72-3.18) and anemia (aOR, 1.83; 95% CI, 1.14-2.88) were at greatest risk of death, whereas those with type 2 myocardial infarction owing to tachyarrhythmia (aOR, 0.83; 95% CI, 0.65-1.06) or coronary mechanisms (aOR, 1.07; 95% CI, 0.17-3.86) were at similar risk of death as patients with type 1 myocardial infarction.

**Conclusions and Relevance:**

In this secondary analysis of a randomized clinical trial, mortality after type 2 myocardial infarction was associated with the underlying etiologic factor associated with oxygen supply-demand imbalance. Most type 2 myocardial infarctions were associated with tachyarrhythmia, with better prognosis, whereas hypoxemia and anemia accounted for one-third of cases, with double the mortality of type 1 myocardial infarction. These differential outcomes should be considered by clinicians when determining which cases need to be managed if patient outcomes are to improve.

**Trial Registration:**

ClinicalTrials.gov Identifier: NCT01852123

## Key Points

**Question:**

What are the clinical outcomes of the different factors associated with oxygen supply-demand imbalance among patients with type 2 myocardial infarction?

**Findings:**

In this secondary analysis of a randomized clinical trial, tachyarrhythmia was the most common factor associated with oxygen supply-demand imbalance, occurring in 55% of all patients with type 2 myocardial infarction, and was associated with better outcomes. Systemic illnesses associated with type 2 myocardial infarction, which presented with anemia, hypoxemia, hypotension, or severe hypertension, were less common, but patients with these illnesses shared similar characteristics and had the highest rates of all-cause death.

**Meaning:**

The underlying factors associated with oxygen supply-demand imbalance among patients with type 2 myocardial infarction may provide useful prognostic information.

## Teaser

This secondary analysis of a randomized clinical trial evaluates the prevalence and outcomes of different factors associated with oxygen supply-demand imbalance among patients with type 2 myocardial infarction.

## Introduction

The definition of myocardial infarction has evolved because of improved sensitivity of cardiac biomarkers.^1,2,3,4,5^ The Fourth Universal Definition of Myocardial Infarction classifies type 1 myocardial infarction as being due to thrombotic occlusion after atherosclerotic plaque rupture or erosion and type 2 myocardial infarction as being due to myocardial oxygen supply-demand imbalance in the context of acute systemic or cardiac illnesses or an underlying coronary mechanism other than plaque rupture.^3^

It is recognized that type 2 myocardial infarction is common^6,7^ and that it is associated with a substantial risk of adverse clinical outcomes,^8,9,10^ with as few as 30% of patients alive at 5 years.^11,12^ Despite this fact, effective strategies for the investigation and management of type 2 myocardial infarction have not been defined.^3,8,13,14^ Type 2 myocardial infarction is a heterogenous condition encompassing multiple factors associated with reduced myocardial oxygen supply (coronary embolism, dissection or vasospasm, hypotension, hypoxemia, and anemia) and increased myocardial oxygen demand (hypertension and tachyarrhythmia).^3,11^ These etiologic factors may confer different risks of adverse clinical outcomes and require distinct treatment strategies.^15^ In this secondary analysis of a multicenter randomized clinical trial,^16^ we report the frequency of these factors associated with oxygen supply-demand imbalance among patients with type 2 myocardial infarction, and we compare their characteristics and outcomes with those of patients with type 1 myocardial infarction.

## Methods

### Study Population and Trial Design

High-STEACS (High-Sensitivity Troponin in the Evaluation of Patients with Suspected Acute Coronary Syndrome; NCT01852123)^16^ was a stepped-wedge cluster randomized clinical trial that evaluated the implementation of a high-sensitivity cardiac troponin I assay (ARCHITECT STAT; Abbott) for consecutive patients with suspected acute coronary syndrome across 10 hospitals in Scotland between June 10, 2013, and March 3, 2016 (statistical analysis plan in Supplement 1). All patients were screened prospectively by the usual care clinician using an electronic form integrated into the clinical pathway, which captured the indication for cardiac troponin testing and presenting symptoms. The Scotland Research Ethics Committee approved the trial. In the High-STEACS trial the intervention (implementation of a high-sensitivity cardiac troponin I assay) was implemented at hospital level; as such, individual patient consent was not required. In this secondary analysis, which was not prespecified, we included trial patients with an adjudicated diagnosis of type 1 or type 2 myocardial infarctions for whom the underlying factors associated with oxygen supply-demand imbalance were recorded. The trial was conducted according to the Consolidated Standards of Reporting Trials (CONSORT) reporting guideline.

### Adjudication of Myocardial Infarction

All patients with high-sensitivity cardiac troponin I concentrations above the sex-specific 99th centile (16 ng/L for women and 34 ng/L for men [to convert troponin I to micrograms per liter, multiply by 0.001])^17^ had their diagnosis adjudicated according to the Fourth Universal Definition of Myocardial Infarction (eMethods in Supplement 2).^3,5^ Myocardial ischemia was defined as objective when there was electrocardiographic evidence of new ischemic changes or subjective when symptoms were present.^18^ Patients with symptoms or signs of ischemia and increased myocardial oxygen demand or decreased supply were defined as having type 2 myocardial infarction.

### Factors Associated With Oxygen Supply-Demand Imbalance

The primary etiologic factor associated with oxygen supply-demand imbalance among patients with type 2 myocardial infarction was adjudicated (eMethods in Supplement 2). Coronary artery dissection, embolism, and vasospasm were grouped as coronary mechanisms. In this secondary analysis, we compared outcomes for all patients with an adjudicated diagnosis of type 1 or type 2 myocardial infarction among clinically relevant subgroups. Patients with myocardial infarction owing to coronary artery plaque rupture and thrombosis, erosion, dissection, embolism, or vasospasm were grouped in a “coronary mechanisms” category. Patients with myocardial infarction in response to an acute systemic illness, presenting with anemia, hypotension, hypoxemia, or severe hypertension, were grouped in a “systemic illnesses” category. Those with primary tachyarrhythmia comprised the “tachyarrhythmias” category. Where admission observations were available, we determined the proportion of patients with single vs multiple factors associated with oxygen supply-demand imbalance using objective criteria as applied by the National Early Warning Score (heart rate >100 beats per minute, oxygen saturation <94%, and systolic blood pressure <120 or >180 mm Hg).^19^

### Study Outcomes

National registries were used to ensure complete follow-up for the study population during a 1-year period after index admission. The primary outcome was all-cause death at 1 year. We evaluated the secondary outcomes of noncardiovascular death, the composite of myocardial infarction or cardiovascular death, its individual components, and length of hospital admission. Secondary outcome events, including the cause of death, were adjudicated by a panel, blinded to the index diagnosis and study phase (eMethods in Supplement 2).

### Statistical Analysis

Statistical analysis was performed from July 7 to 30, 2020. Baseline characteristics were summarized in subgroups of patients according to the factors associated with oxygen supply-demand imbalance. Continuous variables are reported as median (IQR) values. Categorical variables are reported as percentages. Any categorical variables with a frequency of fewer than 5 are reported as less than 5 owing to data protection requirements. Groupwise comparisons were performed using the χ^2^ test or the Kruskal-Wallis test. The frequency of the primary outcome of all-cause death at 1 year was estimated using the Kaplan-Meier estimate for cumulative incidence, with the log-rank test for comparisons. Based on previous observations of an excess of noncardiovascular deaths among patients with type 2 myocardial infarction, we applied competing risk methods when considering the secondary outcomes of myocardial infarction or cardiovascular death and the competing risk of noncardiovascular death.^12^ Univariable logistic regression modeling and multivariable logistic regression modeling were used to examine the association between the factors associated with oxygen supply-demand imbalance and the primary outcome of all-cause death at 1 year with type 1 myocardial infarction as reference group. In the multivariable model, odds ratios were adjusted for variables identified a priori, including age, sex, history of ischemic heart disease, type 1 or 2 diabetes, and kidney impairment.^20,21^ A complete case analysis was used for evaluating logistic regression models. All *P* values were from 2-sided tests and results were deemed statistically significant at *P* < .05. Statistical analysis was performed in R, version 3.5.1 (R Group for Statistical Computing).

## Results

**Figure 1.**
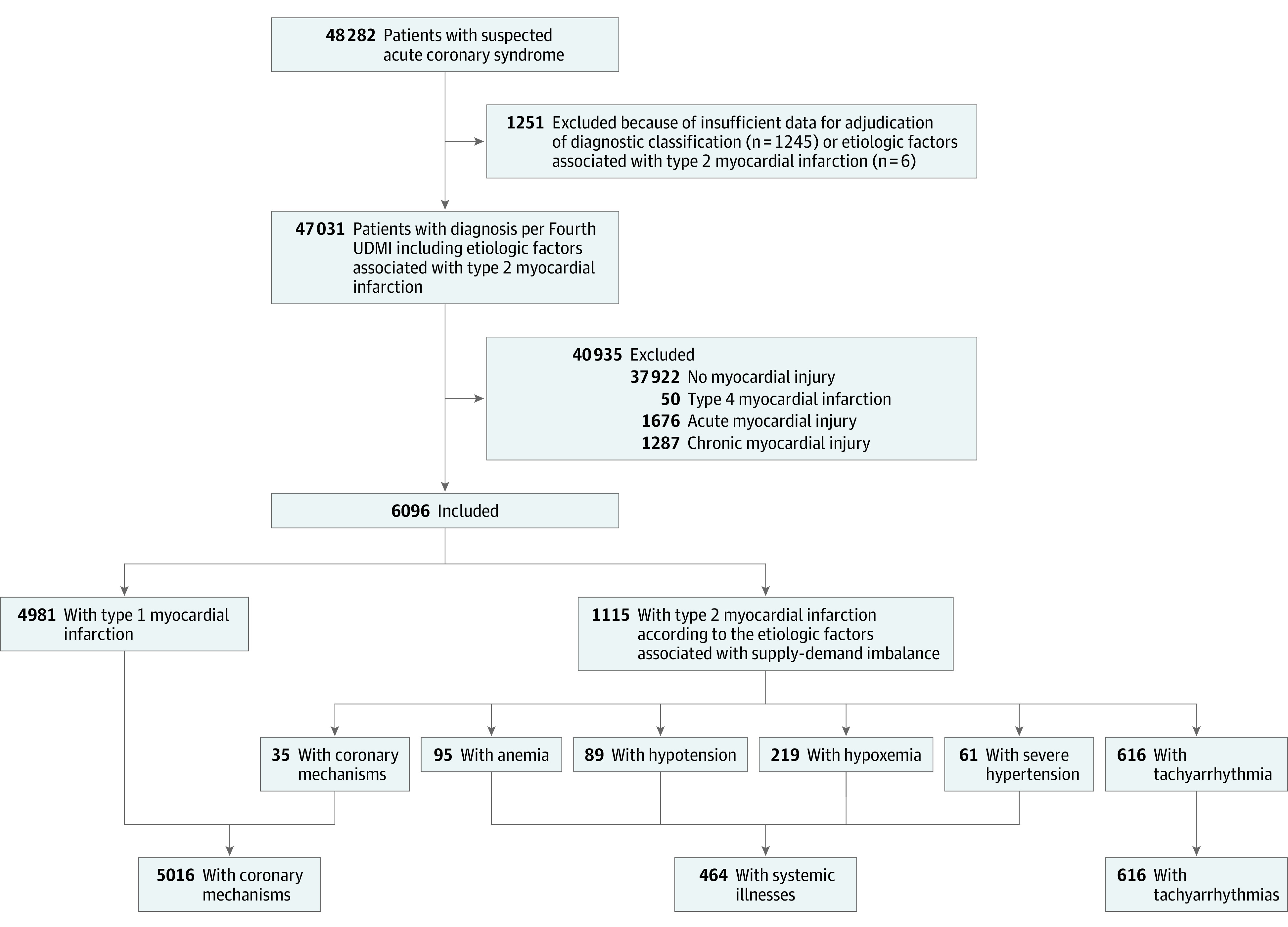
Trial Population UDMI indicates Universal Definition of Myocardial Infarction.

The High-STEACS trial enrolled 48 282 consecutive patients with suspected acute coronary syndrome (22 562 women [47%]; median age, 61 years [IQR, 49-75 years]), of whom 22% (10 360 of 48 282) had high-sensitivity cardiac troponin I concentrations above the 99th centile. Among patients with sufficient information to adjudicate the diagnosis, this was type 1 myocardial infarction for 55% (4981 of 9115) and type 2 myocardial infarction for 12% (1121 of 9115). The primary etiologic factor associated with oxygen supply-demand imbalance was determined for 1115 patients; therefore, this secondary analysis included 6096 patients (2602 women [43%]; median age, 70 years [IQR, 58-80 years]) (Figure 1). Patients with type 2 myocardial infarction were older than those with type 1 myocardial infarction (median age, 77 years [IQR, 67-84 years] vs 68 years [IQR, 57-79 years]) and more likely to be female than male (55% [616 of 1115] vs 40% [1986 of 4981]) (eTable 1 in Supplement 2).

### Factors Associated With Oxygen Supply-Demand Imbalance

Among patients with type 2 myocardial infarction, the primary mechanisms associated with oxygen supply-demand imbalance were tachyarrhythmia (55% [616 of 1115]), hypoxemia (20% [219 of 1115]), anemia (9% [95 of 1115]), hypotension (8% [89 of 1115]), severe hypertension (5% [61 of 1115]), and coronary mechanisms (3% [35 of 1115]) (Table 1). Among those with coronary mechanisms, 51% (18 of 35) had vasospasm, 37% (13 of 35) had dissection, and 11% (4 of 35) had embolism.

**Table 1.** Baseline Characteristics of the Study Population According to Adjudicated Diagnosis and Factors Associated With Type 2 Myocardial Infarction

Characteristic	Patients, No. (%)^a^						
	Type 1 myocardial infarction (n = 4981)	Factors associated with type 2 myocardial infarction					
		Coronary mechanisms (n = 35)	Anemia (n = 95)	Hypotension (n = 89)	Hypoxemia (n = 219)	Severe hypertension (n = 61)	Tachyarrhythmia (n = 616)
Age, median (IQR), y	68 (57-79)	53 (44-62)	78 (70-84)	78 (69-85)	78 (70-85)	80 (69-85)	76 (67-83)
Sex							
Female	1986 (40)	24 (69)	36 (38)	44 (49)	122 (56)	34 (56)	356 (58)
Male	2995 (60)	11 (31)	59 (62)	45 (51)	97 (44)	27 (44)	260 (42)
Presenting symptom^b^							
Chest pain	4061 (89)	33 (94)	76 (86)	51 (61)	117 (59)	43 (81)	423 (75)
Dyspnea	171 (4)	<5 (<14)	8 (9)	8 (10)	56 (28)	<5 (<8)	42 (7)
Palpitation	17 (0.3)	<5 (<14)	<5 (<5)	<5 (<6)	<5 (<2)	<5 (<8)	64 (10)
Syncope	102 (2)	<5 (<14)	<5 (<5)	13 (15)	7 (4)	<5 (<8)	13 (2)
Other	221 (5)	<5 (<14)	<5 (<5)	12 (14)	16 (8)	<5 (<8)	25 (4)
Medical history							
Myocardial infarction	667 (13)	<5 (<14)	16 (17)	13 (15)	32 (15)	7 (12)	91 (15)
Ischemic heart disease	1519 (31)	5 (14)	42 (44)	33 (37)	97 (44)	29 (48)	246 (40)
Cerebrovascular disease	368 (7)	<5 (<14)	14 (15)	14 (16)	30 (14)	5 (8)	72 (12)
Type 1 or 2 diabetes	802 (16)	<5 (<14)	21 (22)	13 (15)	41 (19)	6 (10)	64 (10)
Heart failure hospitalization	792 (16)	<5 (<14)	33 (35)	22 (25)	80 (37)	22 (36)	130 (21)
Kidney impairment	1167 (23)	<5 (<14)	31 (33)	34 (38)	86 (39)	17 (28)	191 (31)
Previous revascularization							
Percutaneous coronary intervention	487 (10)	<5 (<14)	7 (7)	<5 (<6)	10 (5)	6 (10)	66 (11)
Coronary artery bypass grafting	105 (2)	<5 (<14)	<5 (<5)	<5 (<6)	6 (3)	<5 (<8)	18 (3)
Medications at presentation							
Aspirin	1694 (34)	8 (23)	47 (50)	35 (39)	91 (42)	24 (39)	263 (43)
Dual antiplatelet therapy	233 (5)	<5 (<14)	<5 (<5)	<5 (<6)	14 (6)	<5 (<8)	37 (6)
Lipid-lowering therapy	2377 (48)	11 (31)	61 (64)	45 (51)	125 (57)	36 (59)	351 (57)
ACE inhibitor or ARB	1995 (40)	10 (29)	47 (50)	41 (46)	100 (46)	27 (44)	286 (46)
β-Blocker	1598 (32)	8 (23)	43 (45)	31 (35)	80 (37)	21 (34)	303 (49)
Oral anticoagulant	292 (6)	<5 (<14)	16 (17)	12 (13)	28 (13)	9 (15)	105 (17)
Proton pump inhibitor	2037 (41)	8 (23)	64 (67)	45 (51)	102 (47)	34 (56)	292 (47)
Admission electrocardiogram^c^							
Normal	1578 (36)	12 (36)	23 (31)	19 (24)	40 (21)	12 (21)	91 (15)
Myocardial ischemia	1872 (43)	17 (52)	32 (43)	41 (52)	108 (56)	14 (25)	171 (29)
ST-segment elevation	870 (20)	11 (33)	<5 (<5)	<5 (<6)	10 (5)	<5 (<8)	8 (1)
ST-segment depression	865 (20)	5 (14)	30 (41)	26 (33)	73 (38)	10 (18)	134 (23)
T-wave inversion	780 (18)	6 (18)	6 (8)	27 (34)	56 (29)	10 (18)	61 (10)
Physiological parameters, median (IQR)							
Heart rate, beats per minute	76 (65-90)	78 (64-94)	86 (73-100)	82 (66-100)	95 (80-110)	78 (6-89)	118 (87-143)
Systolic blood pressure, mm Hg	141 (124-160)	142 (128-153)	130 (114-146)	104 (90-152)	132 (115-150)	140 (122-162)	130 (111-149)
Respiratory rate, breaths per minute	17 (16-19)	18 (16-20)	18 (16-20)	19 (1-24)	24 (18-30)	18 (17-23)	18 (16-20)
Oxygen saturation, %	97 (96-99)	98 (97-100)	97 (94-99)	96 (94-97)	93 (88-96)	96 (92-97)	97 (95-98)
Laboratory investigations, median (IQR)							
Hemoglobin, g/dL	13.9 (12.4-15.1)	13.8 (13.3-15.1)	7.5 (6.6-8.3)	13.1 (11.4-14.2)	12.8 (11.2-14.0)	12.9 (11.4-14.5)	13.5 (12.2-14.8)
eGFR, mL/min/1.73 m^2^	60 (44-60)	55 (48-60)	42 (31-58)	40 (28-58)	50 (35-60)	57 (41-60)	52 (40-60)
Presentation hs-cTnI, ng/L	102 (33-624)	205 (31-551)	78 (36-302)	81 (35-224)	76 (41-333)	48 (27-133)	40 (18-93)
Peak hs-cTnI, ng/L	855 (104-6775)	1288 (262-4663)	204 (58-1181)	174 (64-727)	148 (51-935)	77 (38-187)	104 (45-433)

Abbreviations: ACE, angiotensin-converting enzyme; ARB, angiotensin receptor blocker; eGFR, estimated glomerular filtration rate; hs-cTnI, high-sensitivity cardiac troponin I.

SI conversion factor: To convert hemoglobin to grams per liter, multiply by 10.0; troponin I to micrograms per liter, multiply by 0.001.

^a^ Cell counts of less than 5 are redacted in line with regulatory approvals.

^b^ Some data on presenting symptoms are missing. The total number of patients by subgroup is type 1 myocardial infarction, 4572; coronary mechanisms, 35; anemia, 88; hypotension, 84; hypoxemia, 198; severe hypertension, 53; and tachyarrhythmia, 567.

^c^ Some electrocardiogram data are missing. The total number of patients by subgroup is type 1 myocardial infarction, 4380; coronary mechanisms, 33; anemia, 74; hypotension, 79; hypoxemia, 187; severe hypertension, 56; and tachyarrhythmia, 605.

### Baseline Characteristics

Patients with coronary mechanisms associated with type 2 myocardial infarction were younger than those with type 1 myocardial infarction (median age, 53 years [IQR, 44-62 years] vs 68 years [IQR, 57-79 years]) (Table 1). In contrast to type 1 myocardial infarction (with 40% [1986 of 4981] women), there were more women than men with type 2 myocardial infarction for most mechanisms associated with oxygen supply-demand imbalance (69% [24 of 35] for coronary mechanisms, 56% [122 of 219] for hypoxemia, 56% [34 of 61] for severe hypertension, and 58% [356 of 616] for tachyarrhythmia) apart from anemia (38% [36 of 95]) and hypotension (49% [44 of 89]). Except for those with coronary mechanisms, patients with type 2 myocardial infarction were older and had higher rates of ischemic heart disease, cerebrovascular disease, heart failure, and kidney impairment compared with patients with type 1 myocardial infarction (Table 1).

### Clinical Presentation

**Figure 2.**
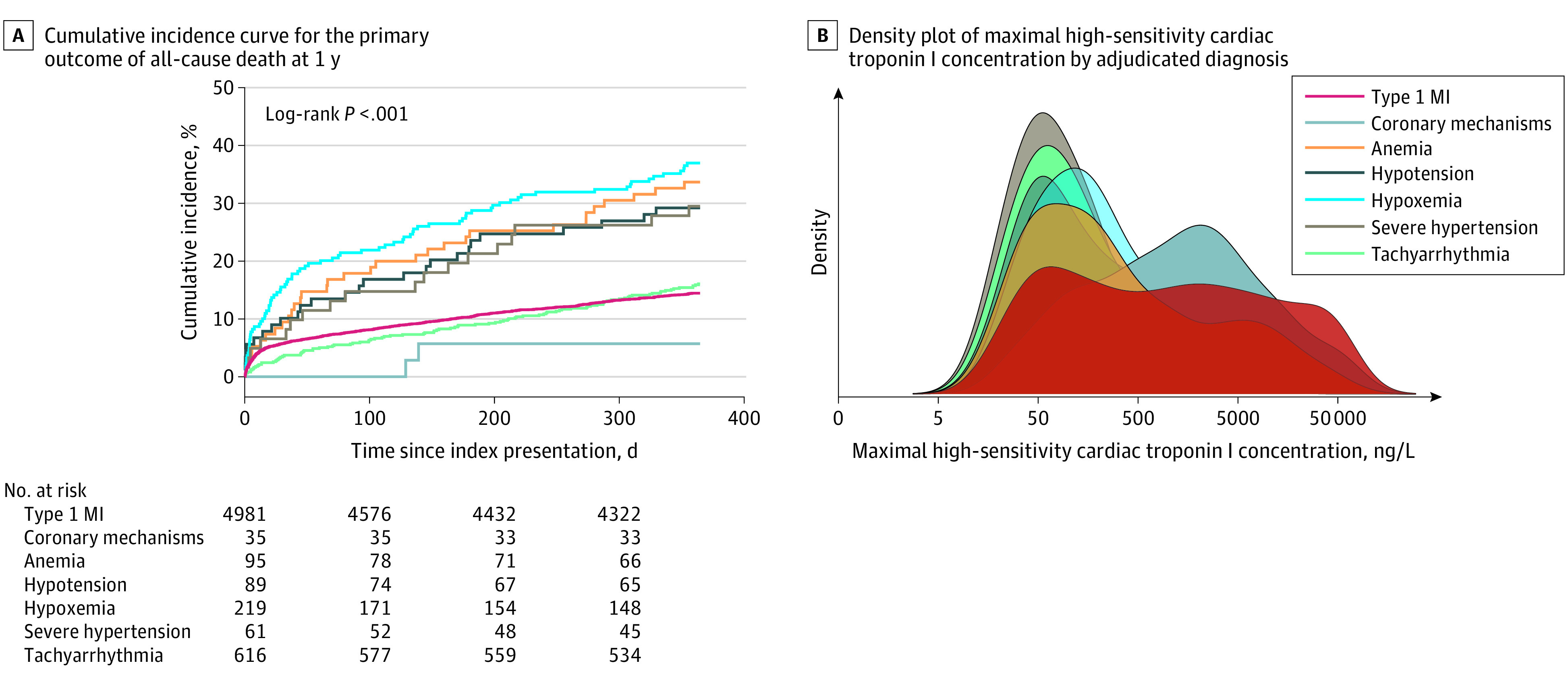
Factors Associated With Oxygen Supply-Demand Imbalance in Type 2 Myocardial Infarction (MI) A, Cumulative incidence curve for the primary outcome of all-cause death at 1 year. B, Kernel density plot showing the distribution of maximal high-sensitivity cardiac troponin I concentrations (ng/L [to convert to micrograms per liter, multiply by 0.001]) according to adjudicated diagnosis and factors associated with oxygen supply-demand imbalance in type 2 myocardial infarction (MI).

Chest pain was the primary presenting symptom for 94% of patients (33 of 35) with a coronary mechanism associated with type 2 myocardial infarction (Table 1). Clinical variables on admission were consistent with the primary factors associated with oxygen supply-demand imbalance; patients with tachyarrhythmia had a high median heart rate (118 beats per minute [IQR, 87-143 beats per minute]) and those with hypoxemia had a low median oxygen saturation level (93% [IQR, 88%-96%]). However, some overlap in clinical measurements was observed; for instance, patients with tachyarrhythmia had lower systolic blood pressure (eFigure 1 in Supplement 2). Median high-sensitivity cardiac troponin I concentrations were highest among patients with a coronary mechanism associated with oxygen supply-demand imbalance (1288 ng/L [IQR, 262-4663 ng/L]) and those with type 1 myocardial infarction (855 ng/L [IQR, 104-6775 ng/L]) (Table 1; Figure 2).

### Clinical Management

Similar to patients with type 1 myocardial infarction, most patients with type 2 myocardial infarction and a coronary mechanism associated with oxygen supply-demand imbalance received medical treatment for suspected acute coronary syndrome (60% [21 of 35] vs 55% [2717 of 4981]) and underwent coronary angiography (63% [22 of 35] vs 59% [2928 of 4981]) (Table 2). Patients with coronary mechanisms associated with type 2 myocardial infarction had similar rates of new aspirin prescriptions compared with patients with type 1 myocardial infarction (49% [17 of 35] vs 45% [2240 of 4981]), while fewer patients with other factors associated with oxygen supply-demand imbalance had commenced antiplatelet therapy. Patients with type 2 myocardial infarction had higher rates of new oral anticoagulant prescriptions compared with those with type 1 myocardial infarction (19% [209 of 1115] vs 3% [129 of 4981]; eTable 2 in Supplement 2), and this was most common among those with tachyarrhythmia (26% [161 of 616]) (Table 2).

**Table 2.** Investigations, Management, and Clinical Outcomes at 1 Year According to Adjudicated Diagnosis and Factors Associated With Type 2 Myocardial Infarction

Characteristic	Patients, No. (%)^a^						
	Type 1 myocardial infarction (n = 4981)	Factors associated with type 2 myocardial infarction					
		Coronary mechanisms (n = 35)	Anemia (n = 95)	Hypotension (n = 89)	Hypoxemia (n = 219)	Severe hypertension (n = 61)	Tachyarrhythmia (n = 616)
Investigations and management							
ACS treatment in the ED	2717 (55)	21 (60)	21 (22)	28 (32)	63 (29)	13 (21)	145 (24)
Medical therapy^b^							
New aspirin	2240 (45)	17 (49)	5 (5)	11 (12)	17 (8)	6 (10)	61 (10)
New P2Y12 inhibitor	3042 (61)	17 (49)	11 (12)	10 (11)	27 (12)	<5 (<8)	65 (11)
New DAPT	2969 (60)	15 (43)	9 (10)	7 (8)	27 (12)	<5 (<8)	54 (9)
New ACE inhibitor or ARB	1577 (32)	10 (29)	9 (10)	5 (6)	17 (8)	5 (8)	57 (9)
New β-blocker	1878 (38)	11 (31)	10 (11)	10 (11)	22 (10)	8 (13)	157 (26)
New lipid-lowering therapy	1764 (35)	9 (26)	10 (11)	5 (6)	11 (5)	<5 (<8)	31 (5)
New oral anticoagulant^c^	129 (3)	5 (14)	<5 (<5)	9 (10)	25 (11)	8 (13)	161 (26)
New proton pump inhibitor	536 (11)	<5 (<14)	14 (15)	8 (9)	10 (5)	<5 (<8)	41 (7)
Coronary investigation or intervention							
Coronary angiography^d^	2928 (59)	22 (63)	<5 (<5)	7 (8)	13 (6)	17 (28)	53 (9)
PCI	2021 (41)	7 (20)	<5 (<5)	<5 (<6)	<5 (<2)	<5 (<8)	7 (1)
Outcomes at 1-y follow-up							
Primary outcome							
All-cause death	720 (14)	<5 (<14)	32 (34)	26 (29)	81 (37)	18 (30)	99 (16)
Secondary outcomes							
Myocardial infarction or cardiovascular death	863 (17)	<5 (<14)	15 (16)	16 (18)	43 (20)	13 (21)	71 (12)
Myocardial infarction	384 (8)	<5 (<14)	<5 (<5)	<5 (<6)	6 (3)	<5 (<8)	27 (4)
Cardiovascular death	479 (10)	<5 (<14)	12 (13)	13 (15)	37 (17)	12 (20)	44 (7)
Noncardiovascular death	241 (5)	0	20 (21)	13 (15)	44 (20)	6 (10)	55 (9)
Length of hospital stay							
Length of stay, median (IQR), d	3 (1-5)	3 (2-4)	5 (2-9)	6 (1-14)	7 (3-14)	4 (1-9)	2 (1-6)

Abbreviations: ACE, angiotensin-converting enzyme; ACS, acute coronary syndrome; ARB, angiotensin receptor blocker; DAPT, dual antiplatelet therapy; ED, emergency department; PCI, percutaneous coronary intervention.

^a^ Cell counts of less than 5 are redacted in line with regulatory approvals. All medications are new prescriptions made during the index hospital admission.

^b^ All medications are new prescriptions made during the index hospital admission.

^c^ Warfarin or direct oral anticoagulant.

^d^ Angiography and revascularization within 30 days of presentation.

### Clinical Outcomes

The primary outcome of all-cause death at 1 year occurred for 15% (720 of 4981) of patients with type 1 myocardial infarction and 23% (258 of 1115) with type 2 myocardial infarction (eTable 2 in Supplement 2). Patients with type 2 myocardial infarction owing to hypoxemia had the highest rates of all-cause death at 1 year (37% [81 of 219]), and those with coronary mechanisms had the lowest rates of all-cause death at 1 year (<14% [<5 of 35]) (Table 2; Figure 2). Patients with tachyarrhythmia also had lower rates of all-cause death (16% [99 of 616]) (Figure 2; eFigure 2 in Supplement 2), similar to patients with type 1 myocardial infarction. In univariable analysis, hypoxemia, anemia, severe hypertension, and hypotension were associated with higher odds of all-cause death at 1 year (eTable 3 in Supplement 2). When accounting for differences in age, sex, and comorbid conditions, the risk of all-cause death at 1 year was twice as high for patients with hypoxemia (adjusted odds ratio [aOR], 2.35; 95% CI, 1.72-3.18) and anemia (aOR, 1.83; 95% CI, 1.14-2.88) compared with patients with type 1 myocardial infarction (eFigure 3 in Supplement 2). Conversely, a trend toward reduction in risk was observed for patients with tachyarrhythmia (aOR, 0.83; 95% CI, 0.65-1.06) and similar risk for those with a coronary mechanism (aOR, 1.07; 95% CI, 0.17-3.86). Patients with multiple etiologic factors associated with oxygen supply-demand imbalance had higher rates of all-cause death at 1 year compared with those with a single etiologic factor (eFigure 4 in Supplement 2).

The rate of myocardial infarction or cardiovascular death at 1 year was lower among patients with type 2 myocardial infarction secondary to tachyarrhythmia (12% [71 of 616]) or coronary mechanisms associated with oxygen supply-demand imbalance (<5% [<5 of 35]) compared with those with type 1 myocardial infarction (17% [863 of 4981]) (Table 2; eFigure 5 in Supplement 2). The rates of noncardiovascular death were 3-fold to 4-fold higher among patients with anemia (21% [20 of 95]), hypoxemia (20% [44 of 219]), and hypotension (15% [13 of 89]) compared with those with type 1 myocardial infarction (5% [241 of 4981]; Table 2; eFigure 5 in Supplement 2). Overall, 8% of patients (384 of 4981) with type 1 myocardial infarction and 4% of patients (42 of 1115) with type 2 myocardial infarction had a subsequent myocardial infarction at 1 year (eTable 2 in Supplement 2). Patients with hypoxemia, hypotension, and anemia had a longer hospital stay (Table 2).

### Subgroup Analysis

Patients with coronary mechanisms associated with type 1 or type 2 myocardial infarction (n = 5016) were younger (median age, 68 years [IQR, 57-79 years]) and more likely to be male (3006 [60%]) compared with those with systemic illness (n = 464; median age, 78 years [IQR, 69-85 years]; 228 men [49%]) or tachyarrhythmia (n = 616; median age, 76 years [IQR, 67-83 years]; 260 men [42%]) (eTable 4 in Supplement 2). Patients with a concomitant systemic illness were more likely to have underlying comorbid conditions. Compared with patients with a systemic illness or tachyarrhythmia, twice as many patients with a coronary mechanism initially received medical therapy for suspected acute coronary syndrome (55% [2738 of 5016] for coronary mechanism vs 27% [125 of 464] for systemic mechanism and 24% [145 of 616] for tachyarrhythmia) (eTable 5 in Supplement 2).

**Figure 3.**
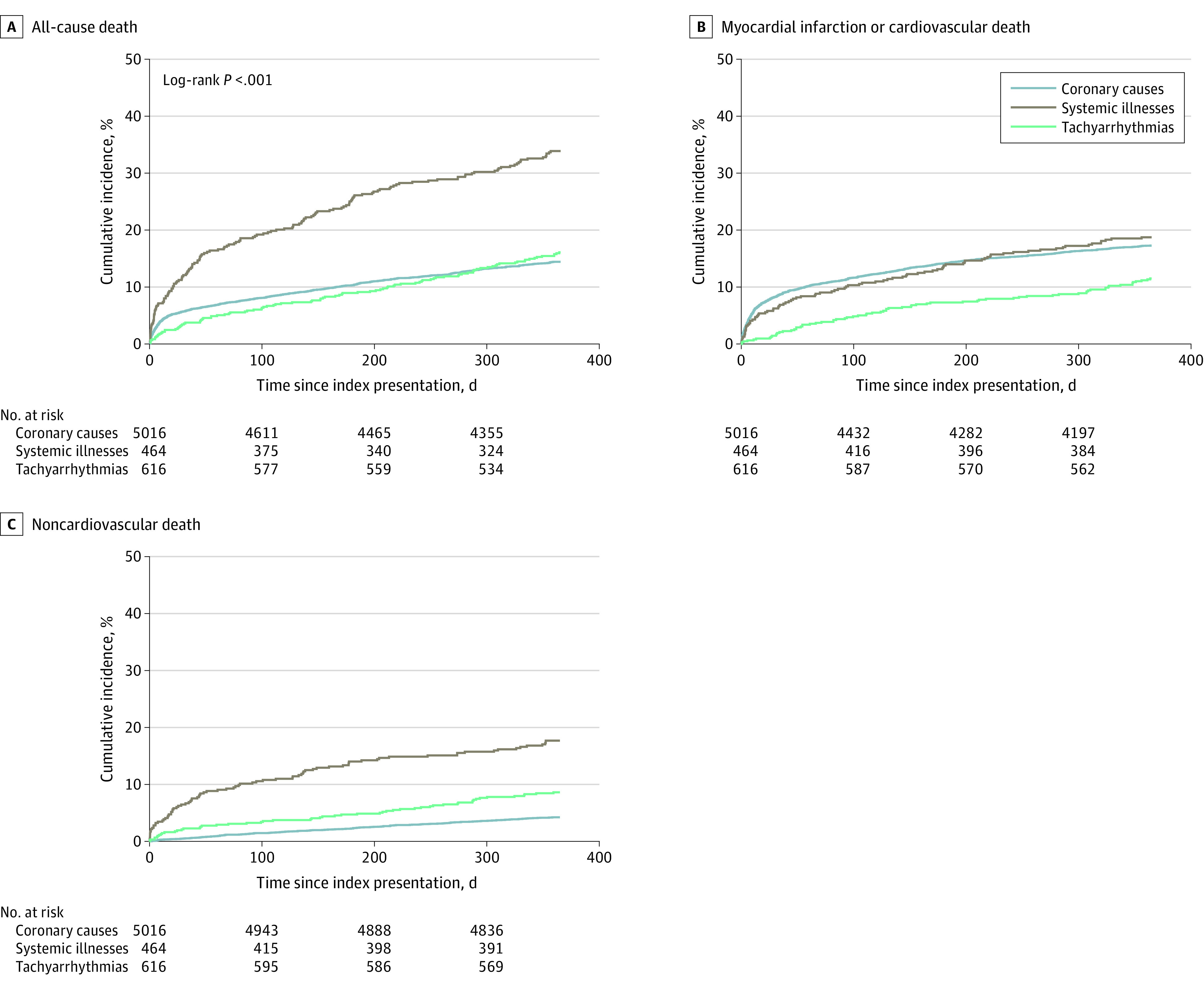
Outcomes According to the Factors Associated With Myocardial Infarction A, Cumulative incidence curves for the primary outcome of all-cause death at 1 year. B, Cumulative incidence curves for the secondary outcomes of myocardial infarction or cardiovascular death. C, Cumulative incidence curves for noncardiovascular death. The “coronary mechanisms” category includes patients with myocardial infarction owing to coronary artery plaque rupture or erosion (type 1) and coronary artery dissection, embolism, or vasospasm (type 2); the “systemic illnesses” category includes patients with myocardial infarction in response to an acute systemic illness, such as anemia, hypotension, hypoxemia, and severe hypertension. Patients with myocardial infarction owing to primary tachyarrhythmia comprise the “tachyarrhythmias” category.

The rate of the primary outcome of all-cause death at 1 year was highest for patients with a systemic illness (34% [157 of 464]) and was double the rate observed for patients with coronary mechanisms (14% [722 of 5016]) and tachyarrhythmia (12% [71 of 616]) (Figure 3; eTable 5 in Supplement 2). Among those with a systemic illness, approximately half the deaths were owing to a cardiovascular factor (47% [74 of 157]) and half were owing to a noncardiovascular factor (53% [83 of 157]). The rate of noncardiovascular death among these patients was substantially higher than among those with a coronary mechanism or tachyarrhythmia (18% [83 of 464] vs 5% [241 of 5016] or 9% [55 of 616], respectively).

## Discussion

Among consecutive hospitalized patients with myocardial infarction, we evaluated the prevalence and outcomes of factors associated with oxygen supply-demand imbalance in type 2 myocardial infarction. We made important observations relevant to practice. First, tachyarrhythmia is the most common factor associated with oxygen supply-demand imbalance, occurring in half of all patients with type 2 myocardial infarction, and is associated with better outcomes. Second, systemic illnesses associated with type 2 myocardial infarction—anemia, hypoxemia, hypotension, or severe hypertension—are less common, but patients with these illnesses as a factor associated with type 2 myocardial infarction share similar characteristics and have the highest rates of all-cause death. Third, while coronary mechanisms are the least prevalent etiologic factors associated with type 2 myocardial infarction, there are major similarities in the presentation, management, and outcomes of patients with these etiologic factors compared with those with type 1 myocardial infarction. Taken together, these findings suggest that for patients with type 2 myocardial infarction, it is important to distinguish the primary cardiac etiologic factors from those associated with hemodynamic stresses of a systemic illness to provide prognostic information.

The increasing frequency of type 2 myocardial infarction^8,11^ and the lack of evidence to guide investigation and treatment for these vulnerable patients poses challenges to clinicians^1,8,9,22^ across specialties, including emergency and general medicine,^23^ intensive care,^24^ and cardiology.^9^ There is marked heterogeneity in referral to cardiology among patients with type 2 myocardial infarction,^25^ and the clinical implications of the different factors associated with type 2 myocardial infarction generate uncertainty.^26,27,28,29^

In this secondary analysis of a prospective randomized clinical trial, we provide new insights into the outcomes of type 2 myocardial infarction according to the factors associated with oxygen supply-demand imbalance. Consistent with previous findings, patients with type 2 myocardial infarction have higher rates of death than those with type 1 myocardial infarction.^5,12,29,30,31,32^ Our analysis demonstrates that this higher rate is owing to an excess in noncardiovascular death, which is confined to patients with type 2 myocardial infarction secondary to the pathophysiological consequences of their systemic illness. These patients have similar risk profiles. In contrast, patients with type 2 myocardial infarction owing to coronary mechanisms or tachyarrhythmia have fewer noncardiovascular events and outcomes similar to those with type 1 myocardial infarction. In a prospective community cohort study, Raphael et al^15^ evaluated rates of all-cause death according to the underlying factors associated with oxygen supply-demand imbalance. They observed that all-cause death was most frequent among patients with hypoxemia and least frequent among those with arrhythmia. Our analysis provides additional insights into the specific cause of death, stratified by etiologic factors.

Patients with hemodynamic stresses, such as anemia, hypotension, hypoxemia, or severe hypertension, have particularly poor clinical outcomes, with a high 1-year mortality that is equally associated with cardiovascular and noncardiovascular factors. The observed substantial excess in noncardiovascular death reflects a poor physiological reserve in response to the systemic illness, underlying frailty, or more severe noncardiovascular comorbid conditions with limited life expectancy.^33,34^ Furthermore, among patients with a systemic illness associated with type 2 myocardial infarction, the excess in fatal outcomes was observed early after the index episode, suggesting that type 2 myocardial infarction is an indicator of illness severity.^24^ Perhaps unsurprisingly, these patients were less likely to undergo cardiac investigations or to receive new treatments. Improving outcomes for this group of patients requires an individualized approach guided by illness severity, comorbid conditions, and the probability that a cardiac investigation may identify treatable disease.

The initial management strategy for patients with type 2 myocardial infarction should primarily address the underlying factors associated with oxygen supply-demand imbalance. Although patients with tachyarrhythmia had more favorable outcomes compared with those with type 1 myocardial infarction or type 2 myocardial infarction owing to a systemic illness, more than 1 in 10 patients with tachyarrhythmia had a further myocardial infarction or died owing to a cardiovascular factor at 1 year. This finding likely reflects the risk of complications from tachyarrhythmia, such as thromboembolism or structural heart disease.^35,36^ The use of antiarrhythmic therapies and anticoagulants could plausibly reduce this risk.

Although coronary mechanisms, such as spontaneous coronary artery dissection, embolism, or vasospasm, were the least prevalent etiologic factor associated with type 2 myocardial infarction, patients with these mechanisms comprise a distinct group. These patients are younger, with lower prevalence rates of comorbid conditions; most present with chest pain and substantially elevated cardiac troponin concentrations similar to those with type 1 myocardial infarction. They are initially managed similarly to patients with type 1 myocardial infarction and undergo emergency coronary angiography. Once the underlying coronary mechanism is defined during angiography, treatments necessarily diverge.^14,37^ The rationale for including these patients in the same category as patients with other factors associated with type 2 myocardial infarction has been questioned,^38^ with the current classification causing confusion among patients and clinicians.

In addition to considering the primary factors associated with oxygen supply-demand imbalance, it is crucial to recognize that patients who have a type 2 myocardial infarction secondary to an acute illness may have more than 1 factor associated with oxygen supply-demand imbalance. Our data support findings from Raphael et al^15^ suggesting that patients with multiple etiologic factors are likely to have worse clinical outcomes. For example, a patient with sepsis from pneumonia may have hypoxia but will also often have tachycardia and hypotension, adding to the magnitude of the overall insult.^39^

Although our knowledge of the clinical characteristics and outcomes among patients with type 2 myocardial infarction continues to evolve, we now require randomized clinical trials to determine the benefits or harms associated with investigation and treatment. Our findings have the potential to inform the selection of patients for future trials. These data suggest that the competing risk of noncardiovascular mortality associated with type 2 myocardial infarction is very likely to reduce the effectiveness of proposed cardiovascular interventions. The Appropriateness of Coronary Investigation in Myocardial Injury and Type 2 Myocardial Infarction (ACT-2) trial is a randomized evaluation of the role of early invasive or computed tomography coronary angiography vs conservative management in all-cause mortality in type 2 myocardial infarction.^40^

### Strengths and Limitations

Our study has several strengths. We enrolled consecutive patients across 10 secondary and tertiary care hospitals, ensuring that our findings are generalizable. Our study population was representative, comprising individuals at low risk and individuals at high risk. Adjudication was informed by clinical information, including medical history, discharge documentation, and investigations. Furthermore, the factors associated with oxygen supply-demand imbalance were documented prospectively.

Our study also has some limitations. We acknowledge that few patients with type 2 myocardial infarction underwent coronary angiography; as such, it is likely that some were misclassified. Although the trial population included consecutive patients with suspected acute coronary syndrome, this may have been associated with the prevalence of type 2 myocardial infarction and the underlying factors associated with oxygen supply-demand imbalance, which may have been different if consecutive hospitalized patients undergoing cardiac troponin testing for any indication had been enrolled.^41^ Data on hemodynamic variables were missing for a proportion of patients, which limits the scope of an analysis of those patients with multiple physiological stressors, and we did not have information on clinical frailty.

## Conclusions

Tachyarrhythmia was found to be the most common etiologic factor associated with type 2 myocardial infarction and was associated with a favorable prognosis compared with patients with oxygen supply-demand imbalance owing to an acute systemic illness. In addition to offering prognostic information, efforts to distinguish systemic factors associated with type 2 myocardial infarction from primary cardiac etiologic factors may facilitate targeted investigation and treatment.

## Notes

Supplement 1.
Trial Protocol and Statistical Analysis Plan

Supplement 2.
eMethods.
**eTable 1.** Characteristics of the Study Population According to Adjudicated Diagnosis (Type 1 and Type 2 Myocardial Infarction) **eTable 2.** Investigations, Management and Clinical Outcomes at 1 Year According to Adjudicated Diagnosis **eTable 3.** Associations of the Etiology of Type 2 Myocardial Infarction and All-Cause Death at 1 Year **eTable 4.** Characteristics of the Study Population According to Etiology Subgroups of Type 1 and Type 2 Myocardial Infarction **eTable 5.** Investigations, Management and Clinical Outcomes at 1 Year According to Etiology Subgroups of Type 1 and Type 2 Myocardial Infarction **eFigure 1.** Panel of Scatter Plots Showing the Distributions of Patients According to Etiology of Supply-Demand Imbalance in Type 2 Myocardial Infarction and Their Clinical Parameters at Presentation **eFigure 2.** Cumulative Incidence Curves for the Primary Outcome of All-Cause Death at 1 Year in Patients With Tachyarrhythmia and Available Admission Electrocardiogram (n = 593), Stratified by Underlying Rhythm on Admission Electrocardiogram **eFigure 3.** Forest Plot Showing Adjusted Odds Ratios (aOR) and 95% CIs for All-Cause Death at 1 Year Stratified by Etiology of Supply-Demand Imbalance as Compared With Type 1 Myocardial Infarction **eFigure 4.** Cumulative Incidence Curves for the Primary Outcome of All-Cause Death at 1 Year Stratified According to the Number of Underlying Etiologies of Supply-Demand Imbalance in Type 2 Myocardial Infarction (Single vs Multiple) **eFigure 5.** Panel Plot of Cumulative Incidence Curves for the Secondary Outcomes of Myocardial Infarction or Cardiovascular Death and Noncardiovascular Death at 1 Year Stratified by the Underlying Etiology of Supply-Demand Imbalance in Type 2 Myocardial Infarction and the Reference Group of Type 1 Myocardial Infarction

Supplement 3.
Nonauthor Collaborators

Supplement 4.
Data Sharing Statement
