# Error in Byline

**DOI:** 10.1001/jamanetworkopen.2023.14903

**Published:** 2023-05-08

In the Original Investigation titled “Assessment of Oxygen Supply-Demand Imbalance and Outcomes Among Patients With Type 2 Myocardial Infarction: A Secondary Analysis of the High-STEACS Cluster Randomized Clinical Trial,” published July 11, 2022, Dr Lee’s name should be listed as Kuan Ken Lee, MD. This article has been corrected.^1^
