# Supplementary material for: Assessment of Oxygen Supply-Demand Imbalance and Outcomes Among Patients With Type 2 Myocardial Infarction: A Secondary Analysis of the High-STEACS Cluster Randomized Clinical Trial
Source: JAMA Netw Open. 2022 Jul 11;5(7):e2220162. doi: 10.1001/jamanetworkopen.2022.20162 (PMC9274319; doi:10.1001/jamanetworkopen.2022.20162)
Supplement: Supplement 1. — Trial Protocol and Statistical Analysis Plan [file jamanetwopen-e2220162-s001.pdf]

Assessment of supply-demand imbalance and outcomes in patients with type 2 myocardial infarction

A secondary analysis of the High-STEACS cluster randomized trial

Anda Bularga, MD,[1] Caelan Taggart, MD,[1] Filip Mendusic, MSc,[1] Dorien M. Kimenai, PhD,[2] Ryan Wereski, MD,[1] Matthew T.H. Lowry, MD,[1] Kuan K. Lee, MD,[1] Amy V. Ferry, PhD,[1] Stacey S. Stewart, BSc,[1] David A. McAllister, MD PhD,[3] Anoop S.V. Shah, MD PhD,[4,5] Atul Anand, MD PhD,[1,2] David E. Newby, MD PhD,[1] Nicholas L. Mills, MD PhD,[1,2] Andrew R. Chapman, MD PhD[1] *on behalf of the High-STEACS Investigators*[§]

[1]BHF Centre for Cardiovascular Science, University of Edinburgh, Edinburgh, EH16 4SA, UK
[2]Usher Institute, University of Edinburgh, Edinburgh, EH16 4UX, UK
[3]Institute of Health and Wellbeing, University of Glasgow, G12 8RZ, UK
[4]Department of Non-communicable Disease, London School of Hygiene and Tropical Medicine, London, WC1E 7HT, UK
[5]Department of Cardiology, Imperial College Healthcare NHS Trust, London, W2 1NY, UK

Author: Dr Anda Bularga
Reviewed by: Dr Andrew R Chapman, Professor Nicholas L Mills

Introduction

The definition of myocardial infarction has evolved due to improvements in the sensitivity of cardiac biomarkers, and our understanding of the underlying etiologies of myocardial injury.[1] The Fourth Universal Definition of Myocardial Infarction is an international consensus document which classifies a type 1 myocardial infarction due to atherosclerotic plaque rupture or erosion with intracoronary luminal thrombosis, and type 2 myocardial infarction due to myocardial oxygen supply-demand imbalance in the context of another medical condition such as tachyarrhythmia, hypotension or hypoxia or an underlying coronary mechanism other than plaque rupture.[2] Type 2 myocardial infarction is common and associated with substantial risk of adverse clinical outcomes, worse than type 1 myocardial infarction, with as few as 30% of patients still alive at five years.[3,4] However, this broad diagnostic term encompasses multiple causes of supply-demand imbalance, which may be associated with different risks of adverse outcomes.[5]

Aims and Objectives

We aim to assess the prevalence and clinical outcomes of different etiologies of supply-demand imbalance related to survival in the High-STEACS (*High*-*S*ensitivity *T*roponin in the *E*valuation of patients with *A*cute *C*oronary *S*yndrome) randomised controlled trial.

Outcomes

The primary outcome of this analysis is all-cause death at 1 year and the secondary outcomes the composite of myocardial infarction and cardiovascular death at 1 year and non-cardiovascular death at 1 year. We will evaluate the length of index hospital admission stratified by the underlying etiology of supply-demand imbalance.

Population

In this analysis we will include all participants enrolled in the High-STEACS stepped wedge cluster randomized controlled trial with an adjudicated diagnosis of type 2 myocardial infarction (n=1,115) as per the Fourth Universal Definition of Myocardial Infarction (evidence of supply-demand imbalance and evidence of ischaemia). In patients with type 2 myocardial infarction the underlying cause of supply-demand imbalance was prospectively adjudicated whilst blinded to outcomes following the index presentation. A consort diagram will illustrate the identification of the study population according to the cause of supply-demand imbalance.

Statistical Analysis

Type 2 myocardial infarction patients will be grouped based on the prospectively adjudicated cause of supply-demand imbalance (anaemia, coronary mechanism, hypotension, hypoxaemia, severe hypertension and tachyarrhythmia). For the purpose of this analysis the etiologies of reduced coronary perfusion such as coronary embolus, coronary dissection or coronary vasospasm are grouped in a coronary mechanism category. Baseline characteristics, clinical observations, investigations and treatments received will be summarized as mean (SD) or median (IQR) as appropriate based on distribution. Baseline characteristics will be stratified by the overall population and in subgroups of underlying etiologies of supply-demand imbalance. The prevalence of causes of supply-demand imbalance (n, %) will be evaluated in this cohort of patient with an adjudicated diagnosis of type 2 myocardial infarction.

Event rates will be calculated for primary and secondary outcomes at 1 year. This will be reported for the overall type 2 myocardial infarction study population and stratified by the cause of demand-supply imbalance.

The risk of the primary outcome of all-cause mortality and the secondary outcomes of myocardial infarction or cardiovascular death and non-cardiovascular death at 1 year stratified by the cause of supply-demand imbalance will be estimated using the cumulative incidence function. Table of numbers at risk will be reported.

Univariable and multivariable logistic regression modelling will be used to examine the association between the etiology of supply-demand imbalance of type 2 myocardial infarction and the primary outcome of all-cause death at 1 year. In the multivariable model odds ratios will be adjusted for relevant covariates identified *à priori:* age, sex, prior history of ischemic heart disease, diabetes mellitus and renal impairment. Statistical analysis will be performed on the NHS Safe Heaven platform using R Studio version 3.5.1 (R Foundation for Statistical Computing, Vienna, Austria).

Expected value of analysis

Type 2 myocardial infarction is common and is associated with poor clinical outcomes. This diagnosis is based on clinical, echocardiographic and biomarker evaluation and it encompasses a range of supply-demand etiologies. There is an urgent need to further understand the clinical characteristics and underlying cause of type 2 myocardial infarction to enable us to support the risk stratification and management of these patients.

References

1. Shah ASV, Anand A, Strachan FE, Ferry AV, Lee KK, Chapman AR, et al. High-sensitivity troponin in the evaluation of patients with suspected acute coronary syndrome: a stepped-wedge, cluster-randomised controlled trial. Lancet. 2018;392:919-928.

2. Thygesen K, Alpert JS, Jaffe AS, Chaitman BR, Bax JJ, Morrow DA, et al. Fourth universal definition of myocardial infarction (2018). Eur Heart J. 2018.

3. Chapman AR, Shah ASV, Lee KK, Anand A, Francis O, Adamson P, et al. Long-Term Outcomes in Patients With Type 2 Myocardial Infarction and Myocardial Injury. Circulation. 2018;137(12):1236-45.

4. Chapman AR, Adamson PD, Shah ASV, Anand A, Strachan F, Ferry AV, et al. High-Sensitivity Cardiac Troponin and the Universal Definition of Myocardial Infarction. Circulation. 2020;242:161-171.

5. Raphael CE, Roger VL, Sandoval Y, Singh M, Bell M, Lerman A, et al. Incidence, Trends and Outcomes of Type 2 myocardial Infarction in a Community Cohort. Circulation. 2020;141:454-463.

Table 1. Patient demographics for the overall type to population and stratified by etiology of supply-demand imbalance

Table 2. Investigation and management of patients with type 2 myocardial infarction stratified by etiology of supply-demand imbalance.

Table 3. Primary and secondary outcomes stratified by etiology of supply-demand imbalance at 1 year.

Figure 1. Consort diagram showing study population

Figure 2. Cumulative incidence plot showing event rates (all-cause mortality and non-cardiovascular mortality) at 1 year stratified by etiology of supply-demand imbalance.

Figure 2. Forest plot showing adjusted Odds Ratio for all-cause mortality and non-cardiovascular death at 1 year following index admission stratified for type 2 myocardial infarction supply-demand imbalance etiology.
