# Supplementary material for: Assessment of Oxygen Supply-Demand Imbalance and Outcomes Among Patients With Type 2 Myocardial Infarction: A Secondary Analysis of the High-STEACS Cluster Randomized Clinical Trial
Source: JAMA Netw Open. 2022 Jul 11;5(7):e2220162. doi: 10.1001/jamanetworkopen.2022.20162 (PMC9274319; doi:10.1001/jamanetworkopen.2022.20162)
Supplement: Supplement 2. — eMethods. eTable 1. Characteristics of the Study Population According to Adjudicated Diagnosis (Type 1 and Type 2 Myocardial Infarction) eTable 2. Investigations, Management and Clinical Outcomes at 1 Year According to Adjudicated Diagnosis eTable 3. Associations of the Etiology of Type 2 Myocardial Infarction and All-Cause Death at 1 Year eTable 4. Characteristics of the Study Population According to Etiology Subgroups of Type 1 and Type 2 Myocardial Infarction eTable 5. Investigations, Management and Clinical Outcomes at 1 Year According to Etiology Subgroups of Type 1 and Type 2 Myocardial Infarction eFigure 1. Panel of Scatter Plots Showing the Distributions of Patients According to Etiology of Supply-Demand Imbalance in Type 2 Myocardial Infarction and Their Clinical Parameters at Presentation eFigure 2. Cumulative Incidence Curves for the Primary Outcome of All-Cause Death at 1 Year in Patients With Tachyarrhythmia and Available Admission Electrocardiogram (n = 593), Stratified by Underlying Rhythm on Admission Electrocardiogram eFigure 3. Forest Plot Showing Adjusted Odds Ratios (aOR) and 95% CIs for All-Cause Death at 1 Year Stratified by Etiology of Supply-Demand Imbalance as Compared With Type 1 Myocardial Infarction eFigure 4. Cumulative Incidence Curves for the Primary Outcome of All-Cause Death at 1 Year Stratified According to the Number of Underlying Etiologies of Supply-Demand Imbalance in Type 2 Myocardial Infarction (Single vs Multiple) eFigure 5. Panel Plot of Cumulative Incidence Curves for the Secondary Outcomes of Myocardial Infarction or Cardiovascular Death and Noncardiovascular Death at 1 Year Stratified by the Underlying Etiology of Supply-Demand Imbalance in Type 2 Myocardial Infarction and the Reference Group of Type 1 Myocardial Infarction [file jamanetwopen-e2220162-s002.pdf]

Supplementary Online Content

Bularga A, Taggart C, Mendusic F, et al; High-Sensitivity Troponin in the Evaluation of Patients with Suspected Acute Coronary Syndrome (High-STEACS) Investigators. Assessment of oxygen supply-demand imbalance and outcomes among patients with type 2 myocardial infarction: a secondary analysis of the High-STEACS cluster randomized clinical trial. *JAMA Netw Open.* 2022;5(7):e2220162. doi:10.1001/jamanetworkopen.2022.20162

eMethods.
eTable 1. Characteristics of the Study Population According to Adjudicated Diagnosis (Type 1 and Type 2 Myocardial Infarction)
eTable 2. Investigations, Management and Clinical Outcomes at 1 Year According to Adjudicated Diagnosis
eTable 3. Associations of the Etiology of Type 2 Myocardial Infarction and All-Cause Death at 1 Year
eTable 4. Characteristics of the Study Population According to Etiology Subgroups of Type 1 and Type 2 Myocardial Infarction
eTable 5. Investigations, Management and Clinical Outcomes at 1 Year According to Etiology Subgroups of Type 1 and Type 2 Myocardial Infarction
eFigure 1. Panel of Scatter Plots Showing the Distributions of Patients According to Etiology of Supply-Demand Imbalance in Type 2 Myocardial Infarction and Their Clinical Parameters at Presentation
eFigure 2. Cumulative Incidence Curves for the Primary Outcome of All-Cause Death at 1 Year in Patients With Tachyarrhythmia and Available Admission Electrocardiogram (n = 593), Stratified by Underlying Rhythm on Admission Electrocardiogram
eFigure 3. Forest Plot Showing Adjusted Odds Ratios (aOR) and 95% CIs for All-Cause Death at 1 Year Stratified by Etiology of Supply-Demand Imbalance as Compared With Type 1 Myocardial Infarction
eFigure 4. Cumulative Incidence Curves for the Primary Outcome of All-Cause Death at 1 Year Stratified According to the Number of Underlying Etiologies of Supply-Demand Imbalance in Type 2 Myocardial Infarction (Single vs Multiple)
eFigure 5. Panel Plot of Cumulative Incidence Curves for the Secondary Outcomes of Myocardial Infarction or Cardiovascular Death and Noncardiovascular Death at 1 Year Stratified by the Underlying Etiology of Supply-Demand Imbalance in Type 2 Myocardial Infarction and the Reference Group of Type 1 Myocardial Infarction

This supplementary material has been provided by the authors to give readers additional information about their work.

© 2022 Bularga A et al. *JAMA Network Open.*

eMethods.

Detailed description of diagnostic adjudication

All patients with hs-cTnI concentrations above the sex-specific 99th centile were classified according to the Third Universal Definition of Myocardial Infarction in use at the time of the trial. The classification was updated in accordance with the Fourth Universal Definition of Myocardial Infarction for pre-specified secondary analyses. The final diagnosis was adjudicated according to a pre-specified list (cardiac diagnoses: acute aortic dissection, acute heart failure, cardiomyopathy, chronic heart failure, hypertensive heart disease, myopericarditis, non-ST segment elevation myocardial infarction, ST-segment elevation myocardial infarction, recent myocardial infarction, tachyarrhythmia, Takotsubo cardiomyopathy or valvular heart disease; non-cardiac diagnoses: acute kidney injury, chronic kidney disease, chronic obstructive pulmonary disease, gastrointestinal bleed, pulmonary embolism, sepsis, or other). Two physicians independently reviewed all clinical information, blinded to study phase, with discordant diagnoses resolved by a third reviewer. Clinical information included the dates and times of presentation and final discharge, the initial emergency department assessment and final discharge letter as documented in the electronic care record, with summaries of all investigations undertaken during the index presentation including the electrocardiogram. The adjudication panel had access to raw clinical information including hemoglobin, creatinine and high-sensitivity cardiac troponin I concentrations, and the reports from invasive coronary angiography. Type 1 myocardial infarction was defined as myocardial necrosis (any hs-cTnI concentration above the 99th centile with a rise and/or fall in hs-cTnI concentration where serial testing was performed) in the context of a presentation with suspected acute coronary syndrome with symptoms or signs of myocardial ischemia on the electrocardiogram. Patients with symptoms or signs of myocardial ischemia and evidence

© 2022 Bularga A et al. *JAMA Network Open.*

of increased oxygen demand or decreased supply (for example, tachyarrhythmia, hypotension, or anemia) secondary to an alternative pathology and myocardial necrosis were defined as type 2 myocardial infarction. The classification of type 2 myocardial infarction also includes patients with coronary vasospasm, embolism or spontaneous dissection without evidence of atherothrombosis related to coronary artery disease. Type 4a myocardial infarction was defined in patients with symptoms or signs of myocardial ischemia following percutaneous coronary intervention where hs-cTnI concentrations were 5-fold greater than the 99th centile or increased further if elevated prior to the procedure. Type 4b myocardial infarction was defined where myocardial ischemia and myocardial necrosis were associated with stent thrombosis documented at angiography. Myocardial injury was defined if hs-cTnI concentrations were above the 99th centile in the absence of any clinical features of myocardial ischemia. All non-ischemic myocardial injury was classified as acute, unless a change of <20% was observed on serial testing or the final adjudicated diagnosis was chronic heart failure or chronic renal failure, where the classification was chronic myocardial injury.

In patients with type 2 myocardial infarction the primary etiology of supply-demand imbalance was classified using the following categories of reduced myocardial perfusion: coronary artery dissection, coronary embolism, coronary vasospasm, sustained bradyarrhythmia, hypotension or shock, hypoxemia and severe anemia (anemia), or increased myocardial oxygen demand: severe hypertension with or without left ventricular hypertrophy (severe hypertension) and sustained tachyarrhythmia (tachyarrhythmia). When defining the primary causes of supply-demand imbalance abnormal levels of relevant clinical parameters were considered, however, strict cut offs for supply-demand clinical variables, such as blood pressure, oxygen saturations or hemoglobin concentrations, were not used as the level at which these lead to demand ischemia will be patient specific.

© 2022 Bularga A et al. *JAMA Network Open.*

Detailed description of outcome linkage and adjudication

It is a statutory requirement that any deaths occurring in Scotland, or outwith Scotland but within the United Kingdom are entered on the Register of Deaths in Scotland within eight days of death. As such, this registry is 100% complete for the study population, which was restricted to those residing in Scotland. This assumes that patients did not emigrate in the year following enrolment. However, the Scottish population is very stable, with low levels of emigration outwith the United Kingdom.

The TrakCare software application (InterSystems Corporation, Cambridge, MA, USA) is an electronic patient record system used at all participating sites, which provided clinical data for all subsequent hospital admissions. All attendances across any participating hospital where cardiac troponin was measured and the hs-cTnI concentration was >99th centile were reviewed and the diagnosis adjudicated. We used the same approach to adjudication as for the index hospital episode with the panel blinded to all cardiac troponin measurements during the index episode and to the study phase.

© 2022 Bularga A et al. *JAMA Network Open.*

eTable 1. Characteristics of the Study Population According to Adjudicated Diagnosis (Type 1 and Type 2 Myocardial Infarction)

	Overall	*Type 1 Myocardial Infarction*	*Type 2 Myocardial Infarction*	*P-value*
Number of patients	6,096	4,981	1,115	
Age, median (IQR), years	70 [58-80]	68 [57-79]	77 [67-84]	<0.001
Women (%)	2,602 (43)	1,986 (40)	616 (55)	<0.001
Men (%)	3,494 (57)	2,995 (60)	499 (45)	<0.001
Presenting symptom				<0.001
Chest pain *(%)*	4,804 (86)	4,061 (89)	743 (72)	
Dyspnea *(%)*	287 (5)	171 (4)	116 (11)	
Palpitation *(%)*	84 (2)	17 (<1)	67 (7)	
Syncope *(%)*	140 (3)	102 (2)	38 (4)	
Other *(%)*	282 (5)	221 (5)	61 (6)	
Past medical history				
Myocardial infarction *(%)*	829 (14)	667 (13)	162 (15)	0.340
Ischemic heart disease *(%)*	1,971 (32)	1,519 (30)	452 (41)	<0.001
Cerebrovascular disease *(%)*	503 (8)	368 (7)	135 (12)	<0.001
Diabetes mellitus *(%)*	948 (16)	802 (16)	146 (13)	0.001
Heart failure hospitalisation *(%)*	1,083 (18)	792 (16)	291 (26)	<0.001
Renal impairment *(%)*	1,528 (25)	1,167 (23)	361 (32)	<0.001
Medications at presentation				
Aspirin *(%)*	2,162 (35)	1,694 (34)	468 (42)	<0.001
Dual antiplatelet therapy *(%)*	297 (5)	233 (5)	64 (6)	0.158
Lipid lowering therapy *(%)*	3,006 (49)	2,377 (48)	629 (56)	<0.001
ACE inhibitor or ARB *(%)*	2,506 (41)	1,995 (40)	511 (46)	<0.001
β-Blocker *(%)*	2,084 (34)	1,598 (32)	486 (44)	<0.001
Oral anticoagulant *(%)*	462 (8)	292 (6)	170 (15)	<0.001
Proton pump inhibitor *(%)*	2,582 (42)	2,037 (41)	545 (49)	<0.001
Previous revascularisation				
PCI *(%)*	583 (10)	487 (10)	96 (9)	0.245
CABG surgery *(%)*	137 (2)	105 (2)	32 (3)	0.150
Admission electrocardiogram				
Normal *(%)*	1,775 (33)	1,578 (36)	197 (19)	<0.001
Myocardial ischemia *(%)*	2,255 (42)	1,872 (43)	383 (37)	0.001
ST-segment elevation *(%)*	906 (17)	870 (20)	36 (4)	<0.001
ST-segment depression *(%)*	1,143 (21)	865 (20)	278 (27)	<0.001
T-wave inversion *(%)*	946 (18)	780 (18)	166 (16)	0.197
Physiological parameters				
Heart rate, median (IQR), beats per minute	79 [66-96]	76 [65-90]	100 [78-126]	<0.001
Systolic blood pressure, median (IQR), mm Hg	139 [120-158]	141 [124-160]	131 [111-151]	<0.001
Respiratory rate, median (IQR), breaths per minute	17 [16-20]	17 [16-19]	18 [16-24]	<0.001
Oxygen saturation, median (IQR), %	97 [95-99]	97 [96-99]	96 [94-98]	<0.001
Laboratory investigations				
Hemoglobin, median (IQR), g/L	137 [122-150]	139 [124-151]	131 [11- 145]	<0.001
eGFR, median (IQR), mL/min/1.73 m^2	58 [42-60]	60 [44-60]	50 [37-60]	<0.001
Presentation hs-cTnI, median (IQR), ng/L	88 [29-478]	102 [33-624]	50 [22-148]	<0.001
Peak hs-cTnI, median (IQR), ng/L	550 [83-4,777]	855 [104-6,775]	125 [48-606]	<0.001

© 2022 Bularga A et al. *JAMA Network Open.*

Between group comparisons are Chi-square test or independent samples *t*-tests. ACE= angiotensin converting enzyme; ARB= angiotensin receptor blockers; CABG= coronary artery bypass grafting; eGFR= estimated glomerular filtration rate; hs-cTnI= high-sensitivity assay; MI= myocardial infarction; and PCI= percutaneous coronary intervention. §Electrocardiogram data available in 5,378/6,096 patients.

© 2022 Bularga A et al. *JAMA Network Open.*

eTable 2. Investigations, Management and Clinical Outcomes at 1 Year According to Adjudicated Diagnosis

	Overall	***Type 1 Myocardial Infarction***	***Type 2 Myocardial Infarction***	***P-value***
Number of patients	6,096	4,981	1,115	
Investigations and management				
ACS treatment in ED (%)	3,008 (49)	2,717 (55)	291 (26)	<0.001
Medical therapy				
New aspirin (%)	2,357 (39)	2,240 (45)	117 (10)	<0.001
New PY12 inhibitor (%)	3,176 (52)	3,042 (61)	134 (12)	<0.001
New DAPT (%)	3,085 (51)	2,969 (60)	116 (10)	<0.001
New ACE inhibitor or ARB (%)	1,680 (28)	1,577 (32)	103 (9)	<0.001
New β-Blocker (%)	2,096 (34)	1,878 (38)	218 (20)	<0.001
New lipid lowering therapy (%)	1,832 (30)	1,764 (35)	68 (6)	<0.001
New oral anticoagulant (%)	338 (6)	129 (3)	209 (19)	<0.001
New proton pump inhibitor (%)	613 (10)	536 (11)	77 (7)	<0.001
Coronary investigation/intervention				
Coronary angiography (%)	3,042 (50)	2,928 (59)	114 (10)	<0.001
PCI (%)	2,038 (33)	2,021 (41)	17 (2)	<0.001
Outcomes at 1-year follow up				
**Primary outcome**				
All-cause death (%)	978 (16)	720 (14)	258 (23)	<0.001
**Secondary outcomes**				
Myocardial infarction or cardiovascular death (%)	1,025 (17)	863 (17)	162 (15)	0.027
Myocardial infarction (%)	426 (7)	384 (8)	42 (4)	<0.001
Cardiovascular death (%)	599 (10)	479 (10)	120 (11)	0.269
Non-cardiovascular death (%)	379 (6)	241 (5)	138 (12)	<0.001
Length of hospital stay				
Length of stay, median (IQR), days	3 [1-5]	3 [1-5]	4 [1-8]	<0.001

Between group comparisons are Chi-square test or independent samples *t*-tests. ACS= acute coronary syndrome; ACE= angiotensin converting enzyme; ARB= angiotensin receptor blocker; DAPT= dual antiplatelet therapy; ED= emergency department; and PCI= percutaneous coronary intervention.

© 2022 Bularga A et al. *JAMA Network Open.*

eTable 3. Associations of the Etiology of Type 2 Myocardial Infarction and All-Cause Death at 1 Year

Etiology of supply-demand imbalance	***Unadjusted odds ratio*** *[95% CI]*
Coronary	0.36 [0.06-1.18]
Anemia	3.01 [1.93-4.60]
Hypotension	2.44 [1.51-3.84]
Hypoxemia	3.47 [2.60-4.61]
Severe hypertension	2.48 [1.39-4.25]
Tachyarrhythmia	1.13 [0.90-1.42]
CI= confidence interval.	

© 2022 Bularga A et al. *JAMA Network Open.*

eTable 4. Characteristics of the Study Population According to Etiology Subgroups of Type 1 and Type 2 Myocardial Infarction

	Coronary causes	*Systemic illnesses*	*Tachyarrhythmias*	*P-value*
Number of patients	5,016	464	616	
Age, median (IQR), years	68 [57-79]	78 [69-85]	76 [67-83]	<0.001
Women (%)	2,010 (40)	236 (51)	356 (58)	<0.001
Men (%)	3,006 (60)	228 (49)	260 (42)	<0.001
Presenting symptom				<0.001
Chest pain *(%)*	4,094 (89)	287 (68)	423 (75)	
Dyspnea *(%)*	171 (4)	74 (17)	42 (7)	
Palpitation *(%)*	17 (0)	<5 (<1)	64 (11)	
Syncope *(%)*	103 (2)	24 (6)	13 (2)	
Other *(%)*	222 (5)	35 (8)	25 (4)	
Past medical history				
Myocardial infarction *(%)*	670 (13)	68 (15)	91 (15)	0.494
Ischemic heart disease *(%)*	1,524 (30)	201 (43)	246 (40)	<0.001
Cerebrovascular disease *(%)*	368 (7)	63 (14)	72 (12)	<0.001
Diabetes *(%)*	803 (16)	81 (17)	64 (10)	0.001
Heart failure hospitalisation *(%)*	796 (16)	157 (34)	130 (21)	<0.001
Renal impairment *(%)*	1,169 (23)	168 (36)	191 (31)	<0.001
Medications at presentation				
Aspirin *(%)*	1702 (34)	197 (42)	263 (43)	<0.001
Dual antiplatelet therapy *(%)*	236 (5)	24 (5)	37 (6)	0.349
Lipid lowering therapy *(%)*	2388 (48)	267 (58)	351 (57)	<0.001
ACE inhibitor or ARB *(%)*	2005 (40)	215 (46)	286 (46)	0.001
β-Blocker *(%)*	1606 (32)	175 (38)	303 (49)	<0.001
Oral anticoagulant *(%)*	292 (6)	65 (14)	105 (17)	<0.001
Proton pump inhibitor *(%)*	2,045 (41)	245 (53)	292 (47)	<0.001
Previous revascularisation				
PCI *(%)*	490 (10)	27 (6)	66 (11)	0.013
CABG surgery *(%)*	105 (2)	14 (3)	18 (3)	0.215
Admission electrocardiogram[§]				
Normal *(%)*	1590 (36)	94 (23)	91 (15)	<0.001
Myocardial ischemia *(%)*	1889 (43)	195 (49)	171 (29)	0.001
ST-segment elevation *(%)*	881 (20)	17 (4)	8 (1)	<0.001
ST-segment depression *(%)*	870 (20)	139 (35)	134 (23)	<0.001
T-wave inversion *(%)*	786 (18)	99 (25)	61 (10)	0.197
Physiological parameters				
Heart rate, median (IQR), beats per minute	76 [65-90]	88 [73-105]	118 [87-143]	<0.001
Systolic blood pressure, median (IQR), mm Hg	141 [124-160]	132 [111-154]	130 [111-149]	<0.001
Respiratory rate, median (IQR), breaths per minute	17 [16-19]	20 [17-25]	18 [16-20]	<0.001
Oxygen saturation, median (IQR), %	97 [96-99]	95 [91-97]	97 [95-98]	<0.001
Laboratory investigations				
Hemoglobin, median (IQR), g/L	139 [124-151]	120 [94-138]	135 [122-148]	<0.001

© 2022 Bularga A et al. *JAMA Network Open.*

eGFR, median (IQR), mL/min/1.73 m^2	60 [44-60]	48 [32-60]	52 [40-60]	<0.001
Presentation hs-cTnI, median (IQR), ng/L	102 [33-623]	72 [37-268]	40 [18-93]	<0.001
Peak hs-cTnI, ng median (IQR), /L	860 [106-6,744]	148 [52-799]	104 [45-433]	<0.001

Between group comparisons are Chi-square test. ACE= angiotensin converting enzyme; ARB= angiotensin receptor blockers; CABG= coronary artery bypass grafting; eGFR= estimated glomerular filtration rate; hs-cTnI= high-sensitivity assay; and PCI= percutaneous coronary intervention. §Electrocardiogram data available in 5,378/6,096 patients.

© 2022 Bularga A et al. *JAMA Network Open.*

eTable 5. Investigations, Management and Clinical Outcomes at 1 Year According to Etiology Subgroups of Type 1 and Type 2 Myocardial Infarction

	Coronary causes	*Systemic illnesses*	*Tachyarrhythmias*	*P-value*
Number of patients	5,016	464	616	
Investigations and management				
ACS treatment in ED	2,738 (55)	125 (27)	145 (24)	<0.001
Medical therapy				
New aspirin	2,257 (45)	39 (8)	61 (10)	<0.001
New PY12 inhibitor	3,059 (61)	52 (11)	65 (11)	<0.001
New DAPT	2,984 (59)	47 (10)	54 (9)	<0.001
New ACE inhibitor or ARB	1,587 (32)	36 (8)	57 (9)	<0.001
New β-Blocker	1,889 (38)	50 (11)	157 (25)	<0.001
New lipid lowering therapy	1,773 (35)	28 (6)	31 (5)	<0.001
New oral anticoagulant	134 (3)	43 (9)	161 (26)	<0.001
New proton pump inhibitor	538 (11)	34 (7)	41 (7)	<0.001
Coronary investigation/intervention				
Coronary angiography	2,950 (59)	39 (8)	53 (9)	<0.001
PCI	2,028 (40)	<5 (<1)	7 (1)	<0.001
Outcomes at 1-year follow up				
**Primary outcome**				
All-cause death (%)	722 (14)	157 (34)	99 (16)	<0.001
**Secondary outcomes**				
Myocardial infarction or cardiovascular death (%)	867 (17)	87 (19)	71 (12)	<0.001
Myocardial infarction (%)	386 (8)	13 (3)	27 (4)	<0.001
Cardiovascular death (%)	481 (10)	74 (16)	44 (7)	<0.001
Non-cardiovascular death (%)	241 (5)	83 (18)	55 (9)	<0.001
Length of hospital stay				
Length of stay, median (IQR), days	3 [1-5]	6 [2-12]	2 [1-6]	<0.001

Values are number (%). Between group comparisons are Chi-square test or independent samples *t*-tests. ACS= acute coronary syndrome; ACE= angiotensin converting enzyme; ARB= angiotensin receptor blocker; DAPT= dual antiplatelet therapy; ED= emergency department and PCI= percutaneous coronary intervention.

© 2022 Bularga A et al. *JAMA Network Open.*

eFigure 1. Panel of Scatter Plots Showing the Distributions of Patients According to Etiology of Supply-Demand Imbalance in Type 2 Myocardial Infarction and Their Clinical Parameters at Presentation. **A.** Systolic blood pressure and oxygen saturation; **B.** Hemoglobin concentration and heart rate.

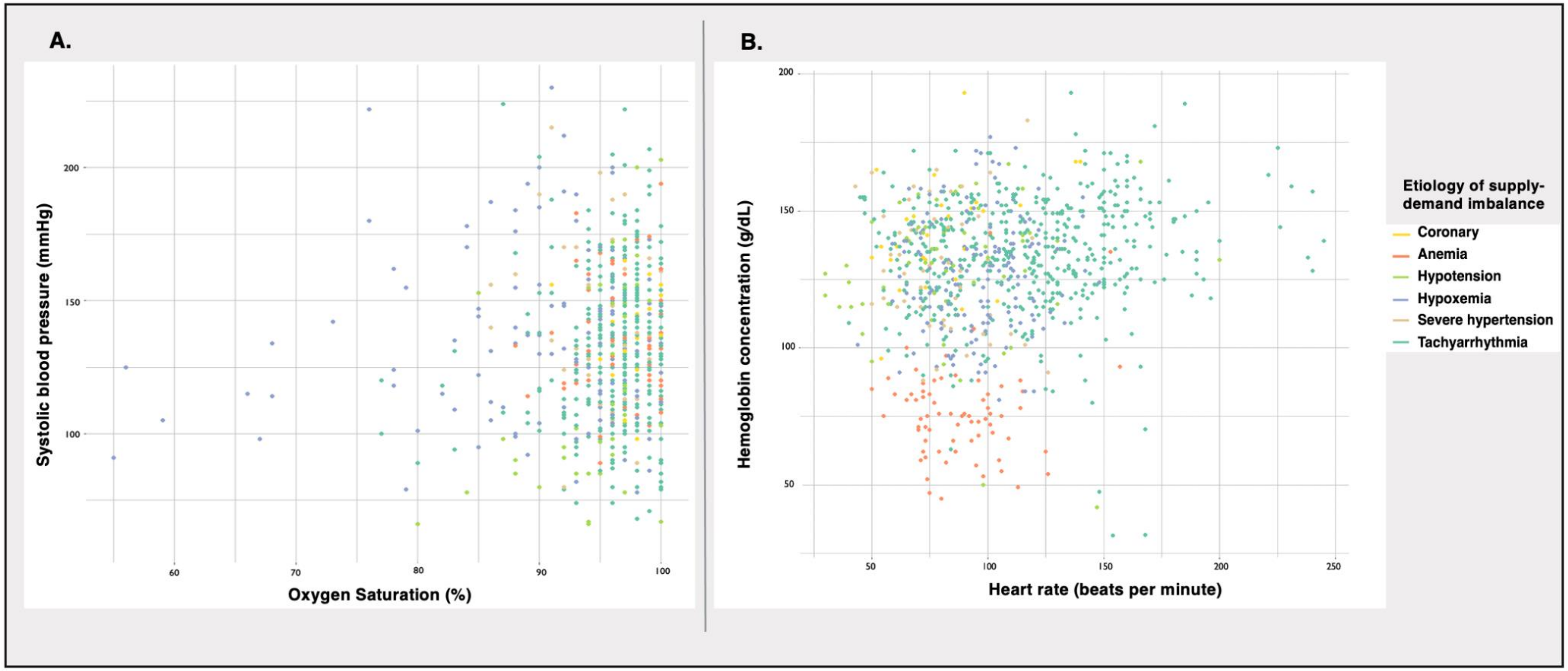

© 2022 Bularga A et al. *JAMA Network Open.*

eFigure 2. Cumulative Incidence Curves for the Primary Outcome of All-Cause Death at 1 Year in Patients With Tachyarrhythmia and Available Admission Electrocardiogram (n = 593), Stratified by Underlying Rhythm on Admission Electrocardiogram. AF/Flutter= atrial fibrillation or atrial flutter; Presumed tachyarrhythmia= patients in whom the admission electrocardiogram showed sinus rhythm, however clinical presentation was suggestive of paroxysmal tachyarrhythmia; SVT= supraventricular tachycardia; VT= ventricular tachycardia.

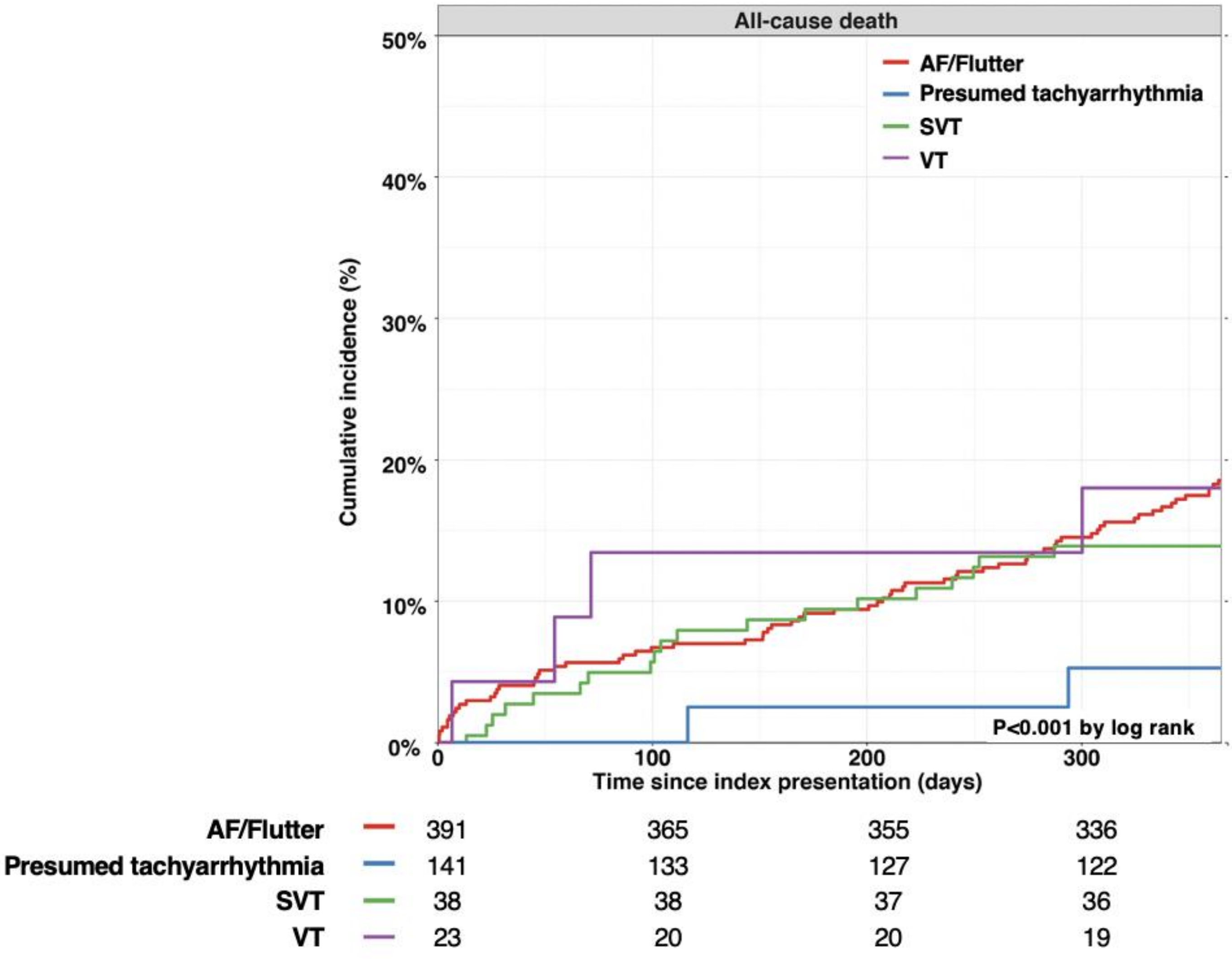

© 2022 Bularga A et al. *JAMA Network Open.*

eFigure 3. Forest Plot Showing Adjusted Odds Ratios (aOR) and 95% CIs for All-Cause Death at 1 Year Stratified by Etiology of Supply-Demand Imbalance as Compared With Type 1 Myocardial Infarction. Model is adjusted for age, sex, prior history of ischemic heart disease, diabetes and renal impairment.

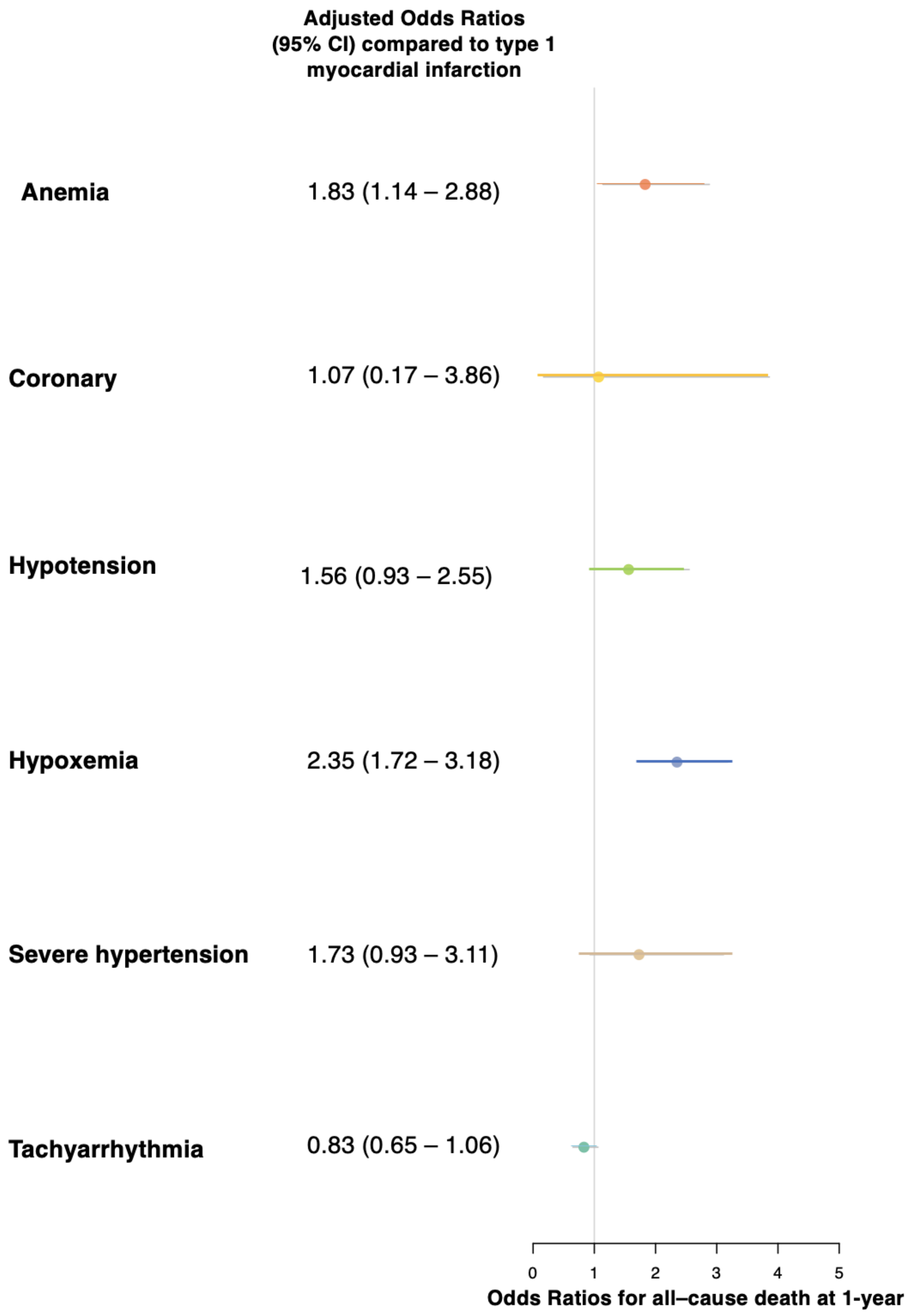

© 2022 Bularga A et al. *JAMA Network Open.*

eFigure 4. Cumulative Incidence Curves for the Primary Outcome of All-Cause Death at 1 Year Stratified According to the Number of Underlying Etiologies of Supply-Demand Imbalance in Type 2 Myocardial Infarction (Single vs Multiple). Admission observations were complete in 58% (650/1,115) of patients, of whom 43% (281/650) had evidence of more than one cause of supply-demand imbalance.

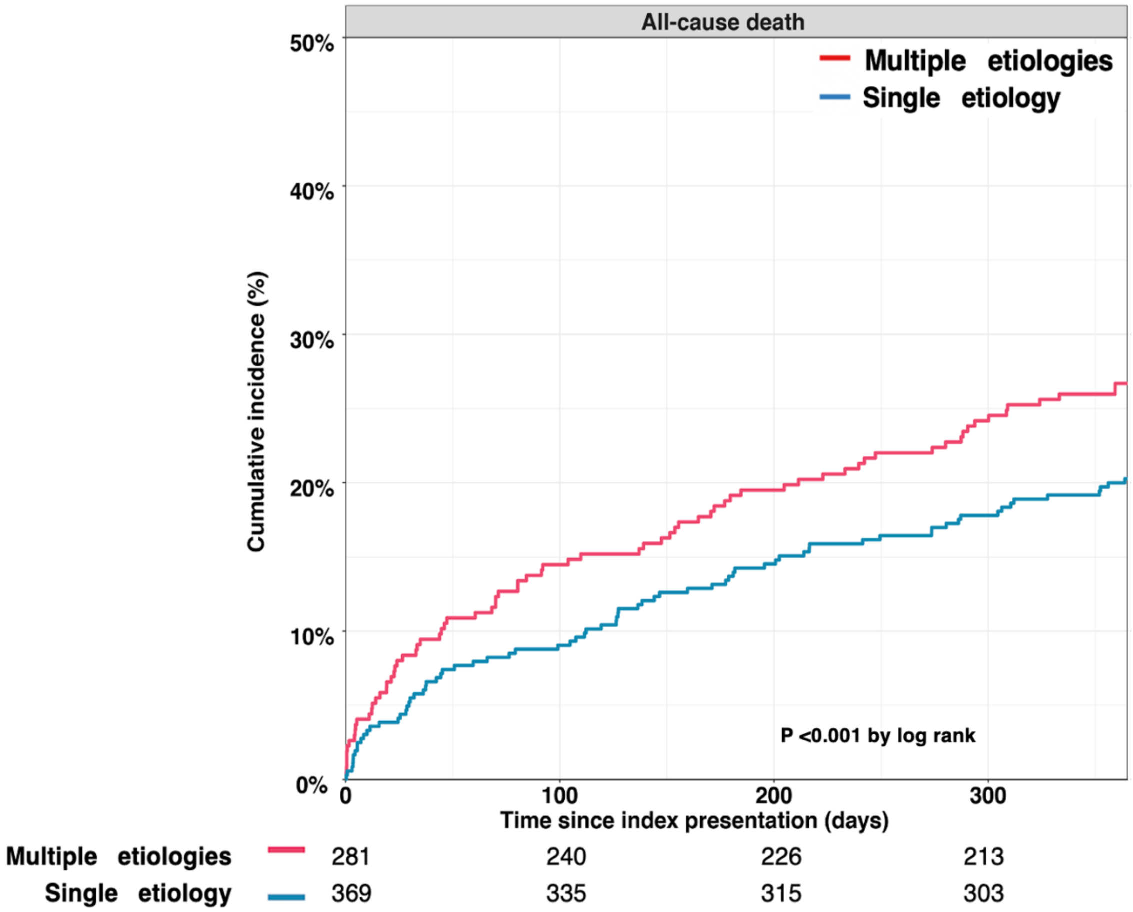

© 2022 Bularga A et al. *JAMA Network Open.*

eFigure 5. Panel Plot of Cumulative Incidence Curves for the Secondary Outcomes of Myocardial Infarction or Cardiovascular Death (**A**) and Noncardiovascular Death (**B**) at 1 Year Stratified by the Underlying Etiology of Supply-Demand Imbalance in Type 2 Myocardial Infarction and the Reference Group of Type 1 Myocardial Infarction

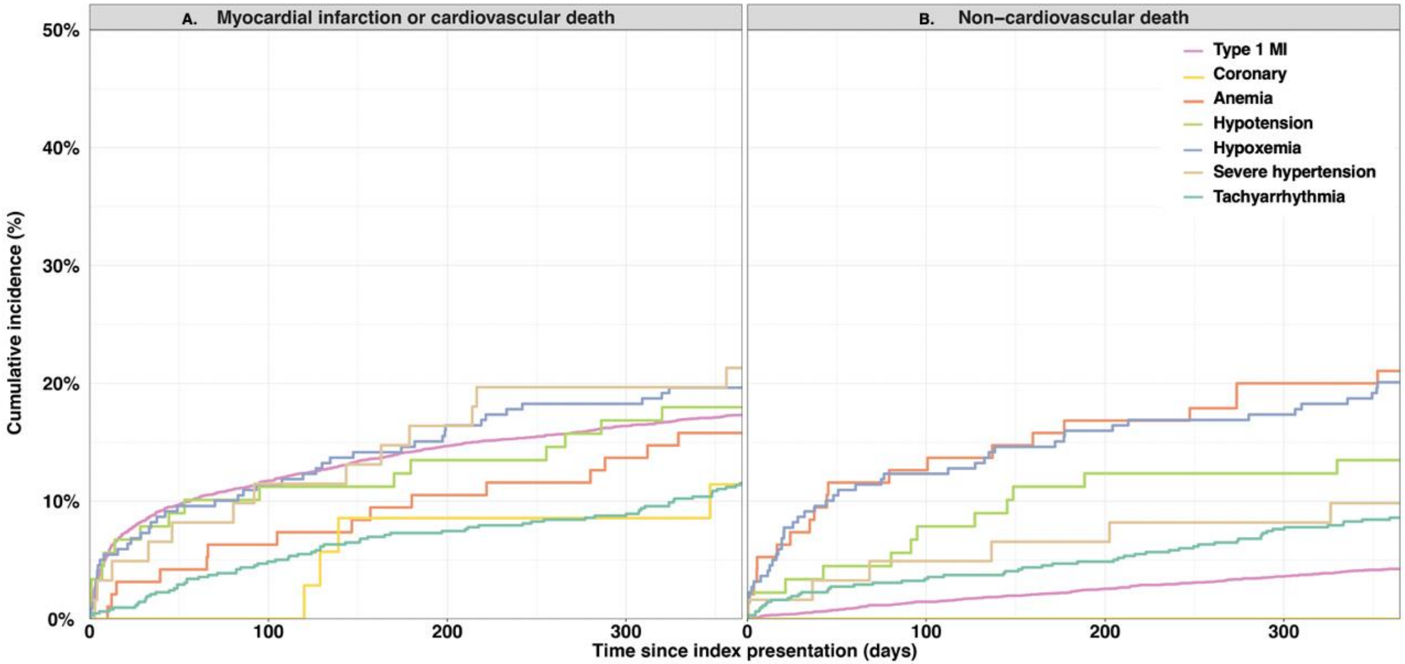

Type 1 MI	4981	4576	4432	4322	4981	4908	4853	4801
Coronary	35	35	32	32	35	35	35	35
Anemia	95	89	85	82	95	83	79	76
Hypotension	89	79	77	74	89	82	78	78
Hypoxemia	219	194	183	179	219	192	184	181
Severe hypertension	61	54	51	49	61	58	57	56
Tachyarrhythmia	616	587	570	562	616	595	586	569

© 2022 Bularga A et al. *JAMA Network Open.*
