# Supplementary material for: Assessment of Oxygen Supply-Demand Imbalance and Outcomes Among Patients With Type 2 Myocardial Infarction: A Secondary Analysis of the High-STEACS Cluster Randomized Clinical Trial
Source: JAMA Netw Open. 2022 Jul 11;5(7):e2220162. doi: 10.1001/jamanetworkopen.2022.20162 (PMC9274319; doi:10.1001/jamanetworkopen.2022.20162)
Supplement: Supplement 3. — Nonauthor Collaborators [file jamanetwopen-e2220162-s003.pdf]

Supplemental Online Content: Nonauthor Collaborators
*First name, last name, and suffix (if applicable) are required and will appear in PubMed.

*Group Name(s): High-Sensitivity Troponin in the Evaluation of Patients with Suspected Acute Coronary Syndrome (High-STEACS) [illegible]							
***First Name and Middle Initial(s)**	***Last Name**	***Suffix (eg, Jr, III)**	Academic Degrees	Institution	Location (city, state/province, country)	Role or Contribution, eg, chair, principal investigator	Group (if more than 1 Group listed in the byline) and/or Subgroup (eg, Steering Committee)
Fiona E	Strachan		PhD	University of Edinburgh	Edinburgh, Scotland, United Kingdom	Trial manager	Trial managers
Christopher	Tuck		BSc	University of Edinburgh	Edinburgh, Scotland, United Kingdom	Trial manager	Trial managers
Dimitrios	Doudesis		MSc	University of Edinburgh	Edinburgh, Scotland, United Kingdom	Researcher	Trial research team
Dennis	Sandeman		BSc	Victoria Hospital Kirkcaldy	Kirkcaldy, Scotland, United Kingdom	Researcher	Trial research team
Philip D	Adamson		PhD	University of Edinburgh	Edinburgh, Scotland, United Kingdom	Researcher	Trial research team
Jack P M	Andrews		PhD	University of Edinburgh	Edinburgh, Scotland, United Kingdom	Researcher	Trial research team
Alastair	Moss		PhD	University of Edinburgh	Edinburgh, Scotland, United Kingdom	Researcher	Trial research team
Mohamed S	Anwar		MD	University of Edinburgh	Edinburgh, Scotland, United Kingdom	Researcher	Trial research team
John	Hung		PhD	University of Edinburgh	Edinburgh, Scotland, United Kingdom	Researcher	Trial research team
Catherine L	Stables		PhD	University of Edinburgh	Edinburgh, Scotland, United Kingdom	Data manager	Data management
Catalina A	Vallejo		PhD	University of Edinburgh	Edinburgh, Scotland, United Kingdom	Researcher	Trial research team
Athanasios	Tsanas		PhD	University of Edinburgh	Edinburgh, Scotland, United Kingdom	Researcher	Trial research team
Lucy	Marshal		BSc	University of Edinburgh	Edinburgh, Scotland, United Kingdom	Researcher	Trial research team
Takeshi	Fujisawa		PhD	University of Edinburgh	Edinburgh, Scotland, United Kingdom	Researcher	Trial research team

Supplemental Online Content: Nonauthor Collaborators
*First name, last name, and suffix (if applicable) are required and will appear in PubMed.

***First Name and Middle Initial(s)**	***Last Name**	***Suffix (eg, Jr, III)**	Academic Degrees	Institution	Location (city, state/province, country)	Role or Contribution, eg, chair, principal investigator	Group (if more than 1 Group listed in the byline) and/or Subgroup (eg, Steering Committee)
Mischa	Hautvast		BSc	University of Edinburgh	Edinburgh, Scotland, United Kingdom	Researcher	Trial research team
Jean	McPherson			University of Edinburgh	Edinburgh, Scotland, United Kingdom	Researcher	Trial research team
Lynn	McKinley			University of Edinburgh	Edinburgh, Scotland, United Kingdom	Researcher	Trial research team
Keith A A	Fox		MD	University of Edinburgh	Edinburgh, Scotland, United Kingdom	Grant applicant	Trial steering committee
Colin	Berry		PhD	University of Glasgow	Glasgow, Scotland, United Kingdom	Grant applicant	Trial steering committee
Simon	Walker		MD	University of Edinburgh	Edinburgh, Scotland, United Kingdom	Biochemistry sub-group committee	Trial steering committee
Christopher	Weir		PhD	University of Edinburgh	Edinburgh, Scotland, United Kingdom	Statistician	Trial steering committee
Ian	Ford		PhD	University of Glasgow	Glasgow, Scotland, United Kingdom	Statistician	Trial steering committee
Alasdair	Gray		PhD	University of Edinburgh	Edinburgh, Scotland, United Kingdom	Researcher	Trial steering committee
Paul O	Collinson		PhD	University of London	London, United Kingdom	Biochemistry sub-group committee	Trial steering committee
Fred S	Apple		PhD	University of Minnesota	Minnesota, United States of America	Researcher	Trial steering committee
Alan	Reid		BSc	University of Glasgow	Glasgow, Scotland, United Kingdom	Biochemistry sub-group committee	Trial steering committee
Anne	Cruikshank		PhD	University of Glasgow	Glasgow, Scotland, United Kingdom	Biochemistry sub-group committee	Trial steering committee
Iain	Findlay		MD	University of Glasgow	Glasgow, Scotland, United Kingdom	Researcher	Trial steering committee

Supplemental Online Content: Nonauthor Collaborators
*First name, last name, and suffix (if applicable) are required and will appear in PubMed.

*First Name and Middle Initial(s)	*Last Name	*Suffix (eg, Jr, III)	Academic Degrees	Institution	Location (city, state/province, country)	Role or Contribution, eg, chair, principal investigator	Group (if more than 1 Group listed in the byline) and/or Subgroup (eg, Steering Committee)
Shannon	Amoils		PhD	British Heart Foundation	United Kingdom	Independent member (steering committee)	Trial steering committee
Donogh	Maguire		PhD	University of Glasgow	Glasgow, Scotland, United Kingdom	Researcher	Trial steering committee
Jennifer	Stevens					Independent member (steering committee)	Trial steering committee
John	Norrie		PhD	University of Edinburgh	Edinburgh, Scotland, United Kingdom	Statistician	Trial steering committee
Jonathan	Malo		MD	University of Edinburgh	Edinburgh, Scotland, United Kingdom	Biochemistry sub-group committee	Biochemistry sub-group committee
Colin M	Fischbacher		PhD	University of Edinburgh	Edinburgh, Scotland, United Kingdom	Data monitoring	Data monitoring committee
Bernard L	Croal		MD	University of Aberdeen	Aberdeen, Scotland, United Kingdom	Data monitoring	Data monitoring committee
Stephen J	Leslie		PhD	University of Aberdeen	Aberdeen, Scotland, United Kingdom	Data monitoring	Data monitoring committee
Catriona	Keerie		BSc	University of Edinburgh	Edinburgh, Scotland, United Kingdom	Trial overview and management	Edinburgh Clinical Trials Unit
Richard A	Parker		BSc	University of Edinburgh	Edinburgh, Scotland, United Kingdom	Trial overview and management	Edinburgh Clinical Trials Unit
Allan	Walker		BSc	University of Edinburgh	Edinburgh, Scotland, United Kingdom	Trial overview and management	Edinburgh Clinical Trials Unit
Ronnie	Harkess		BSc	University of Edinburgh	Edinburgh, Scotland, United Kingdom	Trial overview and management	Edinburgh Clinical Trials Unit
Tony	Wackett		BSc	University of Edinburgh	Edinburgh, Scotland, United Kingdom	Trial overview and management	Edinburgh Clinical Trials Unit
Roma	Armstrong		PhD	University of Glasgow	Glasgow, Scotland, United Kingdom	Trial overview and management	Edinburgh Clinical Trials Unit

Supplemental Online Content: Nonauthor Collaborators
*First name, last name, and suffix (if applicable) are required and will appear in PubMed.

***First Name and Middle Initial(s)**	***Last Name**	***Suffix (eg, Jr, III)**	Academic Degrees	Institution	Location (city, state/province, country)	Role or Contribution, eg, chair, principal investigator	Group (if more than 1 Group listed in the byline) and/or Subgroup (eg, Steering Committee)
Marion	Flood		BSc	University of Glasgow	Glasgow, Scotland, United Kingdom	Trial overview and management	Edinburgh Clinical Trials Unit
Laura	Stirling		BSc	University of Glasgow	Glasgow, Scotland, United Kingdom	Trial overview and management	Edinburgh Clinical Trials Unit
Claire	MacDonald		BSc	University of Glasgow	Glasgow, Scotland, United Kingdom	Trial overview and management	Edinburgh Clinical Trials Unit
Imran	Sadat		BSc	University of Glasgow	Glasgow, Scotland, United Kingdom	Trial overview and management	Edinburgh Clinical Trials Unit
Frank	Finlay		BSc	University of Glasgow	Glasgow, Scotland, United Kingdom	Trial overview and management	Edinburgh Clinical Trials Unit
Heather	Charles		PhD	NHS Lothian Research Governanc	Edinburgh, Scotland, United Kingdom	Research Governance	NHS Lothian Research Governance
Pamela	Linksted		BSc	NHS Lothian Research Governanc	Edinburgh, Scotland, United Kingdom	Research Governance	NHS Lothian Research Governance
Stephen	Young		BSc	NHS Lothian Research Governanc	Edinburgh, Scotland, United Kingdom	Research Governance	NHS Lothian Research Governance
Bill	Alexander		BSc	NHS Lothian Research Governanc	Edinburgh, Scotland, United Kingdom	Research Governance	NHS Lothian Research Governance
Chris	Duncan		BSc	NHS Lothian Research Governanc	Edinburgh, Scotland, United Kingdom	Research Governance	NHS Lothian Research Governance
