# Supplementary material for: Assessment of Oxygen Supply-Demand Imbalance and Outcomes Among Patients With Type 2 Myocardial Infarction: A Secondary Analysis of the High-STEACS Cluster Randomized Clinical Trial
Source: JAMA Netw Open. 2022 Jul 11;5(7):e2220162. doi: 10.1001/jamanetworkopen.2022.20162 (PMC9274319; doi:10.1001/jamanetworkopen.2022.20162)
Supplement: Supplement 4. — Data Sharing Statement [file jamanetwopen-e2220162-s00.pdf]

Data Sharing Statement

Bularga. Assessment of Oxygen Supply-Demand Imbalance and Outcomes Among Patients With Type 2 Myocardial Infarction. *JAMA Netw Open*. Published July 11, 2022. doi:10.1001/jamanetworkopen.2022.20162

Data
Data available: No

Additional Information
Explanation for why data not available: Data can be made available on request.
